# Mapping heterogeneous region- and tissue-specific brain ageing patterns using quantitative MRI

**DOI:** 10.1093/braincomms/fcag010

**Published:** 2026-01-13

**Authors:** Xinjie Chen, Mario Ocampo-Pineda, Po-Jui Lu, Michelle G Jansen, Kwok-Shing Chan, Marcel Zwiers, Joukje M Oosterman, David G Norris, Andre F Marquand, Lester Melie-Garcia, Cristina Granziera, José P Marques

**Affiliations:** Translational Imaging in Neurology (ThINk) Basel, Department of Biomedical Engineering, Faculty of Medicine, University Hospital Basel and University of Basel, 4123 Allschwil, Switzerland; Department of Neurology, University Hospital Basel, 4031 Basel, Switzerland; Research Center for Clinical Neuroimmunology and Neuroscience Basel (RC2NB), University Hospital Basel and University of Basel, 4031 Basel, Switzerland; Donders Institute for Brain, Cognition and Behaviour, Radboud University, 6525 EN, Nijmegen, The Netherlands; Translational Imaging in Neurology (ThINk) Basel, Department of Biomedical Engineering, Faculty of Medicine, University Hospital Basel and University of Basel, 4123 Allschwil, Switzerland; Department of Neurology, University Hospital Basel, 4031 Basel, Switzerland; Research Center for Clinical Neuroimmunology and Neuroscience Basel (RC2NB), University Hospital Basel and University of Basel, 4031 Basel, Switzerland; Translational Imaging in Neurology (ThINk) Basel, Department of Biomedical Engineering, Faculty of Medicine, University Hospital Basel and University of Basel, 4123 Allschwil, Switzerland; Department of Neurology, University Hospital Basel, 4031 Basel, Switzerland; Research Center for Clinical Neuroimmunology and Neuroscience Basel (RC2NB), University Hospital Basel and University of Basel, 4031 Basel, Switzerland; Donders Institute for Brain, Cognition and Behaviour, Radboud University, 6525 EN, Nijmegen, The Netherlands; Athinoula A. Martinos Center for Biomedical Imaging, Charlestown, MA 02129, USA; Department of Radiology, Harvard Medical School, Boston, MA 02115, USA; Donders Institute for Brain, Cognition and Behaviour, Radboud University, 6525 EN, Nijmegen, The Netherlands; Donders Institute for Brain, Cognition and Behaviour, Radboud University, 6525 EN, Nijmegen, The Netherlands; Donders Institute for Brain, Cognition and Behaviour, Radboud University, 6525 EN, Nijmegen, The Netherlands; Donders Institute for Brain, Cognition and Behaviour, Radboud University, 6525 EN, Nijmegen, The Netherlands; Translational Imaging in Neurology (ThINk) Basel, Department of Biomedical Engineering, Faculty of Medicine, University Hospital Basel and University of Basel, 4123 Allschwil, Switzerland; Department of Neurology, University Hospital Basel, 4031 Basel, Switzerland; Research Center for Clinical Neuroimmunology and Neuroscience Basel (RC2NB), University Hospital Basel and University of Basel, 4031 Basel, Switzerland; Translational Imaging in Neurology (ThINk) Basel, Department of Biomedical Engineering, Faculty of Medicine, University Hospital Basel and University of Basel, 4123 Allschwil, Switzerland; Department of Neurology, University Hospital Basel, 4031 Basel, Switzerland; Research Center for Clinical Neuroimmunology and Neuroscience Basel (RC2NB), University Hospital Basel and University of Basel, 4031 Basel, Switzerland; Donders Institute for Brain, Cognition and Behaviour, Radboud University, 6525 EN, Nijmegen, The Netherlands

**Keywords:** brain ageing, multiparametric MRI, normative model, healthy ageing, age modelling

## Abstract

Brain ageing involves microstructural changes that vary across tissue types and even within regions of those tissues, leading to functional and cognitive alterations. Quantitative MRI (qMRI) offers sensitivity to tissue properties, enabling the identification of differential ageing patterns and distinguishing physiological ageing from pathological changes. In this study, we analysed qMRI data from 293 healthy adults (median age: 52; interquartile range: 36–66; age range: 18–79 years). We applied a multiparametric qMRI approach, including longitudinal relaxation rate (*R*_1_), apparent transverse relaxation rate (*R*_2_*) and Quantitative Susceptibility Mapping, to model normal ageing effects on qMRI metrics across regions using second-order polynomial regression, adjusting for sex, education and cognition. Peak ages in turning points derived from quadratic fits were extracted to capture region-specific age-related differences across cortical grey matter, superficial white matter (sWM) and white matter (WM) bundles. According to the results, *R*_1_ showed the most robust age modelling, whereas *R*_2_* and susceptibility presented greater regional variability. Peak ages varied substantially across regions, reflecting the heterogeneity of age-related microstructural differences. Based on quadratic fits, we identified a spatial gradient in qMRI ageing patterns, with earlier peak ages in WM bundles, followed by sWM and culminating in cortical GM. This gradient followed a posterior-to-anterior pattern in the cortex and an inferior-to-superior pattern in WM bundles, consistently observed across all three qMRI metrics. Our study presents exploratory mapping of region- and tissue-specific ageing patterns across brain grey and WM using multiparametric qMRI, offering insights to support future normative healthy ageing research.

## Teaser

X. Chen *et al*. reported that quantitative MRI identified distinct patterns of brain ageing. Analysing data from healthy adults, they showed region-specific age variations and gradients in tissue properties. These findings highlight heterogeneity in healthy brain ageing and provide a reference for distinguishing normal from pathological changes.

## Graphical Abstract

**Graphical Abstract:**
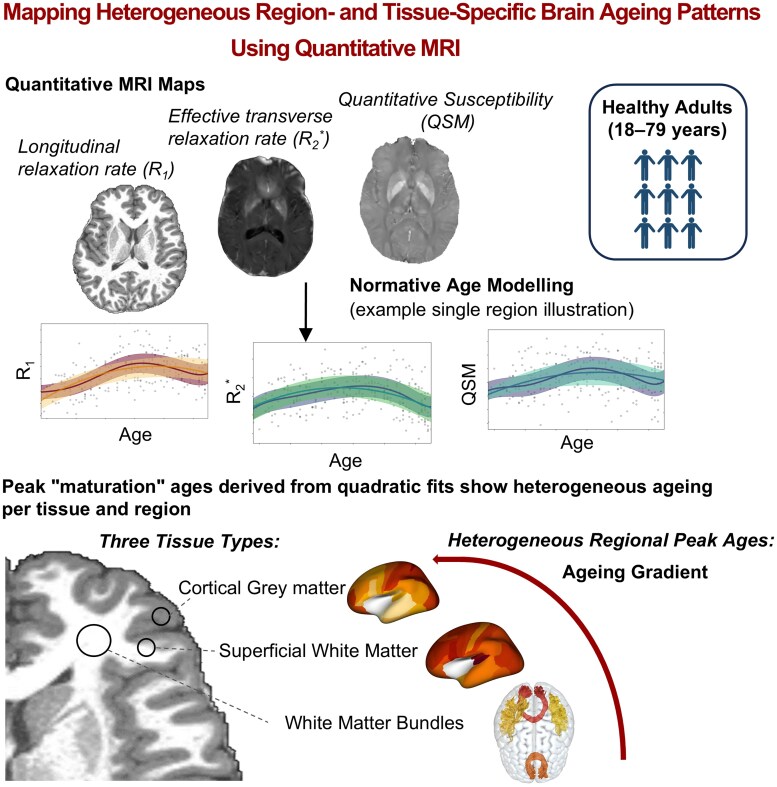

## Introduction

Brain ageing is characterized by fundamental microstructural alterations, including iron accumulation, myelin degradation and brain atrophy. These changes lead to declines, such as cognitive impairment, motor dysfunction and global deterioration of brain function, and ultimately increase the risk of neurological disorders and mortality.^1-3^ Studying tissue changes in the normal ageing process is crucial for explaining the fundamental processes underlying neural ageing in adulthood,^4^ which may help clarify the biological mechanisms underpinning lifespan development.

Brain lifespan development follows established trajectories influenced by functional and anatomical hierarchies across the cortex to the white matter (WM), as described through changes in brain thickness and volume changes.^5-8^ Specifically, cortical grey matter (cGM) development follows a posterior-to-anterior gradient, reaching its early peak thickness in sensory and motor regions during the second and third decades.^9,10^ cGM maturation is characterized by increased synaptogenesis and dendritic complexity together with active synaptic pruning, supporting structural and functional development from infancy through early adulthood, whereas ageing is associated with reduced synaptogenesis and dendritic complexity along with progressive synaptic loss.^11,12^ In contrast, WM development follows a posterior-to-anterior, inferior-to-superior and central-to-peripheral pattern,^13,14^ marked by myelination, axonal elongation and glial proliferation,^15^ which enhances neural signal transmission.^16^ Moreover, superficial white matter (sWM), the WM layer immediately below the cerebral cortex, is composed of short-association fibres that connect adjacent cortical areas and mature more gradually into middle adulthood.^17-19^ Following these development patterns, the brain undergoes age-related structural changes later in life. These age-related changes have been extensively studied using various diffusion-weighted imaging (DWI)-derived metrics and morphometric analyses with *T*_1_-weighted images, from the cortex to WM,^20-23^ providing fundamental insights into normal ageing patterns.

Quantitative MRI (qMRI) extends *T*_1_-weighted imaging by offering quantitative metrics for assessing microstructural changes associated with age.^24^ While *T*_1_-weighted imaging primarily provides qualitative images for anatomical visualization and morphometric measurements,^25^ qMRI can reliably and quantitatively measure a range of tissue properties.^26,27^ Common qMRI metrics include *R*_1_ (longitudinal relaxation rate, *R*_1_ = 1/*T*_1_), *R*_2_* (apparent transverse relaxation rate, *R*_2_* = 1/*T*_2_*) and quantitative susceptibility mapping (QSM). *R*_1_ is primarily influenced by water mobility, tissue density, the size of the compartments and the rate of the exchange processes between both the intracellular and extracellular free water pools with the macromolecular pool.^28,29^ Through this process, *R*_1_ is sensitive to brain microstructural changes associated with myelination in the first decade of life^30-32^ and has been shown to reflect myelin-rich primary sensory areas in cGM with respect to the associative cortex.^33,34^ To a much smaller extent, given the concentration typically present in the brain, *R*_1_ is also sensitive to iron.^35^  *R*_2_* is affected by intra-voxel magnetic field inhomogeneities, thus exhibiting high sensitivity to iron, and is particularly applied in studying tissue properties of deep GM.^36^ However, *R*_2_* also exhibits residual sensitivity to myelin concentration and WM fibre bundle orientation.^37,38^ QSM estimates bulk magnetic susceptibility (per volume), which characterizes how a sample magnetizes in the presence of an external magnetic field. This technique can be used to detect the opposite signs of paramagnetic iron and diamagnetic myelin compared to *R*_2_*.^39,40^ Overall, the integration of multiple qMRI modalities enables a comprehensive understanding of specific tissue characteristics and underlying biophysical mechanisms.

Compared with *T*_1_-weighted imaging-based morphological studies, qMRI-based ageing research investigating microstructural changes remains limited, mainly due to the reliance on advanced hardware (high-count receive coils are essential for achieving a sufficient signal-to-noise ratio and permit acquisition acceleration to avoid lengthy acquisition times) and sequence setups. However, existing studies contributed to a further understanding of the normal brain ageing process by showing insightful microstructural changes with age. Yeatman *et al*.^41^ used *R*_1_ to model lifespan changes in WM tracts, showing that *R*_1_ development rates could predict degeneration rates. In another study, Seiler *et al*.^42^ found that cortical *T*_1_ values decreased and *T*_2_ values increased with age from the second to the eighth decade of life, with *T*_2_ mapping indicating significant global cortical iron deposition as a key process in normal ageing. So far, qMRI techniques have been applied to quantify microstructural differences with age in brain tissues, including WM,^41^ sWM,^43^ cGM^42^ and deep GM,^44^ providing valuable insights into the ageing patterns of different tissue properties. Despite growing interest in qMRI-based ageing studies, coordinated analyses across multiple brain tissues are still scarce. Additionally, cortical investigations have primarily focused on global or lobar-level changes, possibly due to methodological challenges in cortical segmentation (controlling partial volume effects), with finer regional analyses remaining underexplored.^45,46^ Moreover, qMRI-based (and semi-quantitative myelin-sensitive maps) research on the cortical surface has shown its ability to contrast primary cortices (including primary visual, auditory and sensory and motor areas) from association areas.^47^ Despite this body of work, there is minimal research on the age trajectories in these regions. While morphometric studies have revealed distinct ageing patterns across these regions,^48,49^ qMRI can further characterize microstructural changes and their relationship to functional specialization, providing additional insight into brain ageing. There is, therefore, a need for regionally specific, multi-tissue qMRI studies to better understand the heterogeneous ageing patterns across the brain, and particularly from early to late adulthood when these changes are more subtle.

To address the current knowledge gaps, this study aimed to: (i) integrate multiple qMRI metrics (*R*_1_, *R*_2_* and QSM) to comprehensively characterize age-related microstructural changes across brain regions, leveraging their complementary sensitivities to different tissue properties; (ii) identify region- and tissue-specific age-related differences across multiple brain structures (cGM, sWM and WM bundles); (iii) then explore potential brain development and ageing patterns in adulthood. We hypothesized that qMRI-based brain ageing patterns would vary across regions and tissue types, especially with these differences also appearing across functional subdivisions in the cortex. Moreover, different qMRI metrics (*R*_1_, *R*_2_* and QSM) were expected to capture distinct aspects of these processes, providing complementary insights into the microstructural basis of brain ageing.

## Materials and methods

### Study population

This study included 295 healthy participants from the published The Advanced BRain Imaging on ageing and Memory (ABRIM) cohort,^50^ whose demographics, MRI and behaviour data acquisition and preprocessing steps have been previously reported.^51^ This cohort has been previously used to study normative ageing trajectories in deep GM regions.^44^ However, two subjects were excluded due to incompatibility with the processing pipeline (auto-segmentation), resulting in a final sample of 293 subjects. Exclusion criteria included any medical condition that significantly affects brain and cognitive health, including current psychiatric and abnormal neurological conditions, substance use disorders and a history of other major health conditions that could affect cognition.^51^ The educational levels are categorized according to the International Standard Classification of Education (ISCED)^52^ into low, medium and high, while the global cognitive levels are assessed using the Montreal Cognitive Assessment (MoCA).^53^ The study complied with the Helsinki Declaration, received ethical approval (CMO 2014/288; ECSW 2017-3001-46), and all participants gave written informed consent. The cohort's demographic characteristics are reported in Table 1.

**Table 1:** Participant demographics

Description	Column 2 heading
Participant, *n*	293
Female/male, %	53.9/46.1
Median (IQR) age, years	52 (36–66)
Educational level, *n*	
Low	36
Median	90
High	149
Median (IQR) MoCA, scores	52 (26–29)
Age group distribution, *n*	
18–30 years	46
31–40 years	45
41–50 years	47
51–60 years	52
61–70 years	52
71–79 years	51

The educational levels are classified according to the ISCED system into three categories: low, medium, and high. IQR: interquartile range (Q1–Q3); *n*: number; MoCA: Montreal cognitive assessment.

### Image acquisition

The imaging protocols included magnetization prepared two rapid acquisition gradient echoes (MP2RAGE) and multi-shell diffusion and multi-echo gradient recalled echo (ME-GRE) sequences. Images were acquired with a 3T MRI system Magnetom Prisma (Siemens Healthcare, Erlangen, Germany). Acquisition parameters are detailed in Table 2.

**Table 2:** MRI acquisition parameters

Sequence	Parameter	
MP2RAGE-T1	Resolution (mm³)	1 × 1 × 1
	S/P	176
	TR/TI1/TI2 (ms)	6000/700/2400
	FA1,FA2 (°)	6, 6
	ST (min)	7:32
ME-GRE-T2, QSM	Resolution (mm³)	0.8 × 0.8 × 0.8
	TR/TE1/ΔTE (ms)/nE	44/6.14/4/9
	ST (min)	9.25
DWI	Resolution (mm³)	1.8 × 1.8 × 1.8
	B values	0/1250/2500
	Orientation	11/86/85
	ST (min)	9:39

MP2RAGE: magnetization prepared 2 rapid acquisition gradient echoes, ME-GRE: multi-echo gradient recalled echo, QSM: quantitative susceptibility mapping, DWI: diffusion-weighted imaging, S/P: slice/partitions, TR: repetition Time, TI1: inversion Time 1, TI2: inversion time 2, TE1: echo time 1, ΔTE: delta echo time, nE: number of echo. FA: flip-angle(s), ST: scan time. Orientation: the number of diffusion-encoding directions.

### Image preprocessing

#### Data preprocessing

Preprocessing steps were conducted to calculate *R*_1_, *R*_2_* and susceptibility maps. Specifically, MP2RAGE sequences were preprocessed using in-house scripts for *R*_1_ mapping,^54^ while ME-GRE sequences were used to reconstruct *R*_2_* and susceptibility maps with susceptibility mapping pipeline tool for phase images toolbox (1.2.2.4).^55^ DWIs were preprocessed using QSIprep (0.18.0)^56^ for deriving WM bundles. The preprocessing details have been extensively described in previous publications,^51,57^ and also updated in Supplementary Method 1.

#### Cortical parcellation and white matter bundle identification

Cortical segmentation was conducted using FreeSurfer (version 6.0)^58^ with MP2RAGE images.^59^ Cortex was parcellated into 41 Brodmann areas (BAs) using the refined PALS-B12 Brodmann atlas.^60^ The pyAFQ^61^ pipeline from QSIprep was applied to identify 22 WM bundles, including callosum forceps minor (FA), callosum forceps major (FP), and the following bilateral tracts: arcuate (ARC), posterior arcuate fasciculus (pARC), thalamic radiation (ATR), cingulum cingulate (CGC), corticospinal (CST), inferior fronto-occipital fasciculus (IFO), inferior longitudinal fasciculus (ILF), superior longitudinal fasciculus (SLF), vertical occipital fasciculus (VOF) and uncinate (UNC) (see details in Supplementary Method 1).

#### Quantitative mapping across brain structure

Quantitative surface maps were reconstructed by projecting qMRI values onto brain surfaces using FreeSurfer. qMRI values were extracted from the middle layers of cGM and sWM to mitigate partial volume effects. Average qMRI metrics (*R*_1_, *R*_2_* and susceptibility) were computed for each BA, averaging two hemispheres. For WM bundles-based analysis, only the central 70% of the central fibre tract was analysed to minimize partial volume effects, and qMRI measurements were extracted using the Sherbrooke Connectivity Imaging Lab Python dMRI processing toolbox^62^ (for a more detailed description of the procedures, also see details in Supplementary Method 1).

### Statistical analysis and age-related modelling

#### Polynomial quadratic regression model

To facilitate the interpretation of the different ageing patterns per region of interest (ROI) across cGM, sWM and WM bundles, the age-related variation on qMRI metrics was assessed employing a polynomial quadratic regression (PR) model:


(1) $$\mathrm{qMR}I_{\mathrm{ROI}}=\beta_{0\mathrm{ROI}}+\beta_{\mathrm{ROI}}*\mathrm{Age}+\beta_{2\mathrm{ROI}}*\mathrm{Ag}e^{2}+\beta_{3\mathrm{ROI}}*\mathrm{Sex}+\beta_{4\mathrm{ROI}}*\mathrm{Education}+\beta_{5\mathrm{ROI}}*\mathrm{MoCA}$$

This model included age, age², sex, MoCA score and educational level (with medium educational level used as the reference category) as covariates. *β*₁_ROI_ and *β*_2ROI_ are the coefficients of the linear and quadratic terms, respectively. A likelihood-ratio test was used to confirm the superiority of the quadratic term over the linear term.

#### Peak-age estimation

The turning point of the quadratic qMRI age curve, indicating the maximum of age-related tissue property variation and its standard error (SE) were calculated from the PR model using the principles of error propagation (2–3). We performed 10 000 bootstrap resamples to enhance the robustness of the peak-age estimation


(2) $$\mathrm{Ag}e_{\mathrm{peak}}=\text{–}\beta_{\mathrm{ROI}}/\left(2*\beta_{\mathrm{ROI}}\right)$$

(3) $$SE_{\mathrm{peak}}\;=\;\sqrt{{\left(\frac{SE_{\beta_{1\mathrm{ROI}}}}{2\times \beta_{2\mathrm{ROI}}}\right)}^{2}+{\left(\frac{\beta_{1\mathrm{ROI}}\times SE_{\beta_{2\mathrm{ROI}}}}{2\times \beta_{2\mathrm{ROI}}^{2}}\right)}^{2}}$$

Note that when the quadratic coefficient was non-zero, the peak age was determined at the maximum or minimum of the quadratic curve. If the quadratic coefficient was not significant in the likelihood-ratio test, the peak age was not computed.

#### Model evaluation

To ensure model stability and facilitate interpretability, we employed a polynomial model including linear and quadratic age terms. This choice was motivated by the fact that our cohort included only healthy adults, where differences between quadratic and alternative modelling approaches (such as exponential saturation functions or beta splines) are expected to be minimal in terms of explained variance (EXPV), since the most pronounced divergences between these models are expected during childhood and adolescence, when iron accumulation and myelin maturation occur more rapidly.^63^ To assess the robustness and interpretability of the PR model and to evaluate the risk of it being an oversimplified model of the data in this age range, we further compared its performance with that of a more flexible cubic B-spline model. The B-spline model was conducted using the splines package in R, with knots placed at the 25th, 50th and 75th percentiles of age (a commonly used heuristic approach to distribute knots evenly across the predictor range). This placement strikes a balance between flexibility and smoothness while avoiding overfitting. Both models were trained and evaluated using a 5-fold cross-validation framework, ensuring that the same cross-validation splits were used for both models to allow a fair comparison. Model performance was assessed using out-of-sample EXPV,^64^ calculated from the best-performing fold.

To compare the performance of the models, we used the EXPV ratio (B-spline/PR) to quantify their relative ability to capture the variance in age fitting. If the out-of-sample EXPV Ratio is >1, it suggests that the B-spline model captures more variability than the polynomial regression model. Conversely, if the EXPV ratio is <1, it indicates that the B-spline model does not outperform the polynomial regression model in explaining variance. If the EXPV ratio approaches 1, it means a high alignment with the models’ fitting. In both scenarios, we can conclude that metrics extracted from the simple polynomial model (such as the peak age) are interpretable within this age range and does not result from a model feature.

### Age-related regional covariance

Pearson correlation matrices were calculated using pairwise complete observations to explore intra- and inter-regional covariance and age-related influences within cGM and sWM. Correlation matrices were used to assess whether the regional qMRI measurements were independent or formed networks of joint maturation, myelination and iron deposition in cGM and sWM, which were computed using $q\mathrm{MR}I_{\mathrm{RO}I_{i}}$ versus $q\mathrm{MR}I_{\mathrm{ROI}j}$, where qMRI stands for quantitative metric (separately performed for *R*_1_, *R*_2_* and QSM) and $\mathrm{RO}I_{i}$ stands for the ROI in cGM and sWM. A similar analysis was performed for $Zq\mathrm{MR}I_{\mathrm{RO}I_{i}}$ versus $Zq\mathrm{MR}I_{\mathrm{ROI}j}$, where ZqMRI was related to the *Z* score of a metric after accounting for age, sex, education and cognition using the [Eq. 1]. While the first analysis identified regions with similar ageing time courses, the second analysis explored regions with shared covariance independently of ageing. The average correlation coefficients were all Fisher-Z transformed before calculation to ensure accuracy and reliability.

Additionally, multiple comparisons were corrected by the Benjamini–Hochberg method.

## Results

### *R*_1_, *R*_2_* and quantitative susceptibility mapping distribution patterns

#### Cortical grey matter

**Figure 1:**
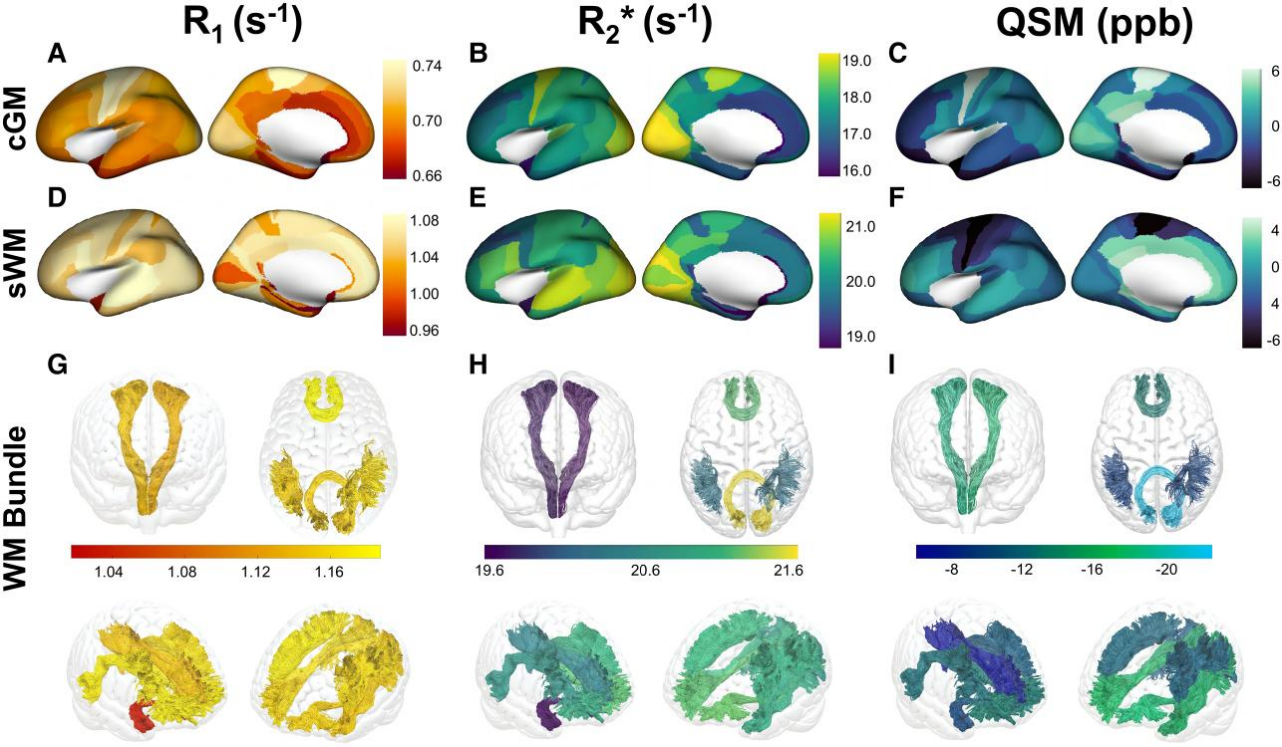
**Mapping regional averages. (A–C)** Surface maps of cGM for *R*_1_, *R*_2_* and susceptibility, respectively. **(D–F)** Surface maps of superficial sWM. **(G–I)** Tractography WM bundles for the same metric. Outliers more than three times the median were excluded before calculating regional averages based on brain parcellation, to account for potential vessel artefacts or segmentation errors. The colour bars in each panel represent the distribution range of average metric values across different brain regions, with warmer colours indicating higher values and cooler colours indicating lower values. Displayed values reflect bilateral averages. QSM: quantitative susceptibility mapping, cGM: cortical grey matter, sWM: superficial white matter, WM: white matter.

The analysis showed the characteristic distribution of *R*_1_ across cortical areas (see Fig. 1): (i) the primary cortices, particularly the primary sensory cortices including the primary visual cortex (V1, BA 17), the primary somatosensory cortex (S1, BA 1, 2, 3), the primary motor (M1, BA4) and the primary auditory cortex (A1, BA 41, 42), exhibited higher *R*_1_ relaxation rates compared to the mean *R*_1_ across cGM regions. (ii) The associative cortices including the prefrontal cortex (BA 9, 10, 11, 46), temporal association areas (BA 20, 21, 22, 37), parietal association cortex (BA 5, 7), occipital association cortex (BA 18, 19), temporal pole (BA 38) and anterior cingulate cortex (BA 24, 33), displayed lower *R*_1_ relaxation rates ranging from 0.66–0.70. (iii) The highest and lowest regional *R*_1_ mean values were observed in BA 4 (primary motor cortex) and BA 25 (part of the associative, anterior cingulate cortex), respectively. For detailed distributions of average *R*_1_ values across different ROIs, refer to Fig. 1A, D, and G.

Our results also showed different distribution patterns of *R*_2_* and susceptibility values across cGM regions (Fig. 1B and C, respectively) compared to *R*_1_: (i) In the primary cortices, the primary motor cortex (BA 4) and primary visual cortex (BA 17, 18) showed higher average values in both *R*_2_* and susceptibility across all cortical regions. Notably, increased susceptibility values were observed in the prefrontal cortex (BA 33), primary auditory cortex (BA 42), and posterior cingulate cortex (BA 23). (ii) The associative cortices displayed intermediate increased *R*_2_* and susceptibility values across the regions, with no extreme responses observed. (iii) Among the regions, the lowest *R*_2_* of 15.04 (± 1.06) s^−1^ was noted in the anterior cingulate cortex (BA 33), while the highest *R*_2_* of 19.96 (± 0.95) s^−1^ were observed in the occipital cortex (BA 18). Similarly, the lowest susceptibility of −3.99 (± 1.73) ppb was noted in the temporal pole (BA 38), and the highest susceptibility values of 8.37 (± 2.33) ppb were found in the primary motor cortex (BA 4).

#### Superficial white matter

sWM regions were localixed based on their spatial correspondence with cortical BAs, and are therefore reported using the overlying cortical labels (Fig. 1D–F): (i) *R*_1_, *R*_2_* and susceptibility in sWM sensory cortices, unlike in cGM areas, did not show the highest qMRI values with respect to remaining regions. (ii) For the associative cortices, especially in the lateral part of the frontal lobe, sWM showed increased *R*_1_, *R*_2_* and susceptibility values. Conversely, in the interior frontal lobe, there was an increase in *R*_1_ and susceptibility values while *R*_2_* was decreased. (iii) Across all the regions, we observed that *R*_2_* in sWM showed the highest and lowest values in the same regions as in cGM, specifically in BA 18 (18.70 ± 1.14) s^−1^ and BA 33 (21.13 ± 0.94) s^−1^, respectively. Interestingly, BA 4 showed the lowest susceptibility of −5.59 (± 2.42) ppb in sWM, in contrast to cGM, which had the highest value.

#### White matter bundles

The *R*_1_, *R*_2_* and susceptibility values analysis showed distinct patterns across bundles (See Fig. 1G–I). (i) For different clusters of bundles, FA and FP showed high *R*_1_ and *R*_2_* values as commissural tracts. Among association tracts, particularly the left inferior fronto-occipital fasciculus (IFOL) showed high *R*_2_* (21.24 ± 1.12 s^−1^) while the low susceptibility (−16.76 ± 2.32 ppb). The left cingulum cingulate and the right cingulum cingulate (CGCR) showed the highest susceptibility values but lower *R*_2_*. Conversely, CST demonstrated lower values in all metrics. (ii) The highest *R*_1_ value was observed in FA (1.19 ± 0.05 s^−1^), while the lowest was in the left uncinate (1.02 ± 0.06 s^−1^). For *R*_2_*, the highest value was in FP (21.61 ± 1.40 s^−1^), with the lowest in the left corticospinal tract (19.65 ± 1.03 s^−1^). In QSM, the CGCR had the highest value (−4.97 ± 2.87 ppb), whereas the FP had the lowest (−21.23 ± 3.32 ppb).

Details of the regional measurement were provided in Supplementary Table 1.

### Age model fitting in *R*_1_, *R*_2_* and quantitative susceptibility mapping

**Figure 2:**
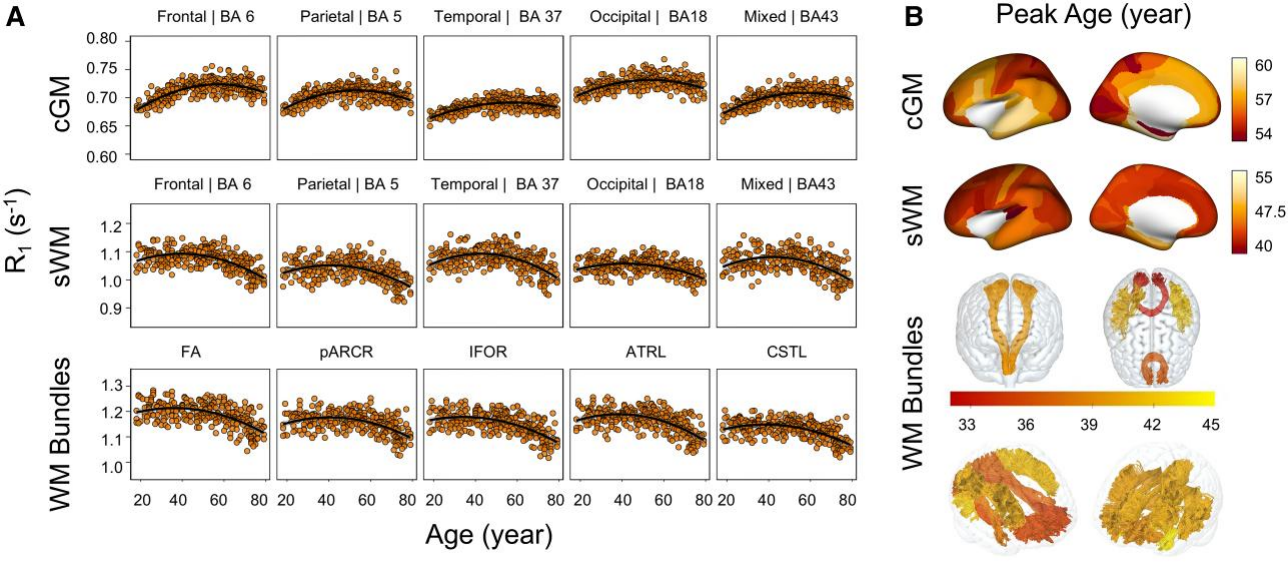
**Modelling *R*_1_ as a function of age. (A)** Representative regions across five different lobes (frontal, parietal, temporal, occipital and mixed—shown from left to right) in the cGM (first row), sWM (second row), and WM bundles (third row). Scatter plots show individual participants (*N* = 293), with quadratic polynomial regression models (solid lines) fitted separately for each region. Shaded areas indicate 95% confidence intervals based on bootstrap resampling (*N* = 10 000). Linear and quadratic models were compared using likelihood-ratio tests, and quadratic fits were generally preferred (*P* > 0.05). The significance of the age and age^2^ effects was assessed using *t*-tests on the regression coefficients. Across regions, both age and age^2^ terms were highly significant (Age: *t* = 4.67–13.30; age^2^: *t* = −11.69 to −6.45; all *P* < 0.001); (**B)** the peak ages for the corresponding *R*_1_ quadratic age models, encoded on the inflated brain for cGM and sWM and in the respective WM bundles. The colour bar represents the peak-age colour coding for each subregion. Displayed values reflect bilateral averages. cGM: cortical grey matter, sWM: superficial white matter, WM: white matter, BA: Brodmann area.

The results showed significant quadratic age dependencies in *R*_1_ across all structures: (i) In cGM, BA 8 in the superior frontal lobe showed the highest *R*² in fitting age effects (*R*^2^ = 0.49, *P* < 0.001); (ii) in sWM, BA 4 in the primary motor cortex showed the highest *R*^2^ in fitting age effects (*R*^2^ = 0.42, *P* < 0.001); (iii) for WM bundles, the IFOL connecting the frontal and occipital lobes, showed the strongest age fitting with the highest *R*^2^ (*R*^2^ = 0.43, *P* < 0.001). Age-related *R*_1_ models in example regions representing five cortical lobes and tract categories were shown in Fig. 2A.

**Figure 3:**
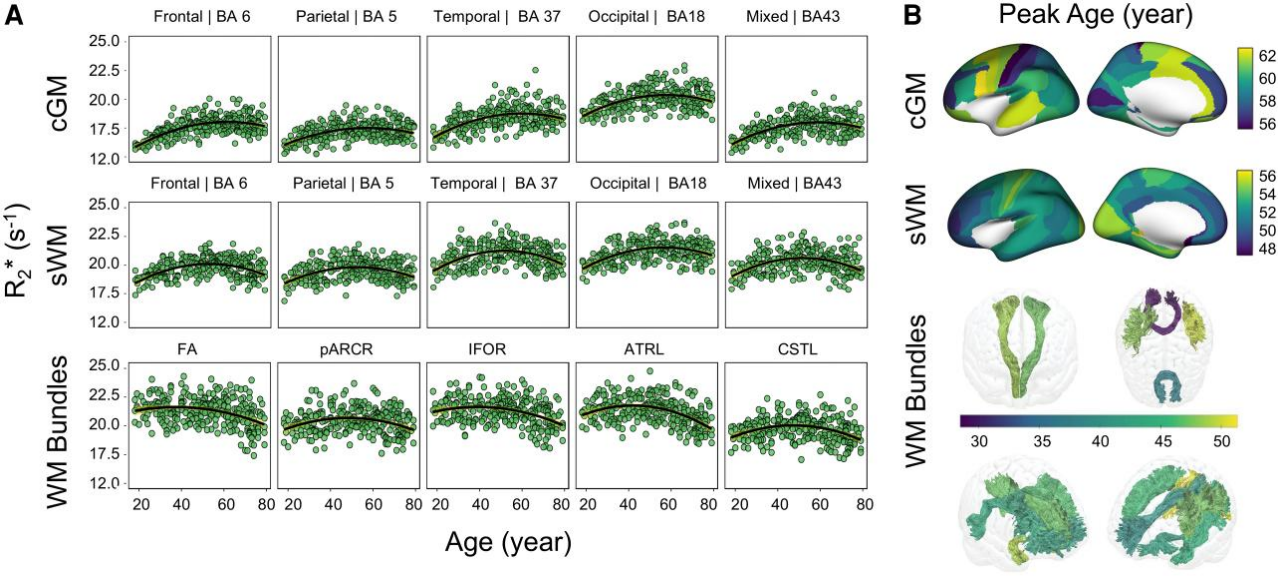
Modelling *R*_2_* as a function of age. (A) Representative regions across five different lobes (frontal, parietal, temporal, occipital and mixed—shown from left to right) in the cGM (first row), sWM (second row) and WM bundles (third row). Scatter plots show individual participants (*N* = 293), with quadratic polynomial regression models (solid lines) fitted separately for each region. Shaded areas indicate 95% confidence intervals based on bootstrap resampling (*N* = 10 000). Model fits were compared to linear alternatives using likelihood-ratio tests. Linear and quadratic models were compared using likelihood-ratio tests, and quadratic fits were generally preferred (*P* > 0.05). The significance of the age and age^2^ effects was assessed using t-tests on the regression coefficients. Across regions, both age and age^2^ terms were significant (Age: *t* = 2.65–9.23, all *P* < 0.01; Age^2^: *t* = −8.78 to −3.60; all *P* < 0.001); (**B)** the peak ages for the corresponding *R*_2_* quadratic age models, encoded on the inflated brain for cGM and sWM and in the respective WM bundles. The colour bar represents the peak-age colour coding for each subregion. Displayed values reflect bilateral averages. cGM: cortical grey matter, sWM: superficial white matter, WM: white matter, BA: Brodmann area.

For *R*_2_*, (i) quadratic age dependencies were significant in most cortical regions except for BA 29 and 26 in cGM, where strong linear dependencies were nevertheless detected. BA 6, part of the premotor cortex, showed the strongest quadratic ageing effect (*R*² = 0.39, *P* < 0.001). (ii) In sWM, the highest *R*^2^ in fitting age effects was noted in BA 4 (*R*² = 0.29, *P* < 0.001), consistent with the findings observed in *R*_1_. (iii) The left thalamic radiation showed the strongest age fitting across WM bundles (*R*^2^ = 0.23, *P* < 0.001) (see Fig. 3A).

**Figure 4:**
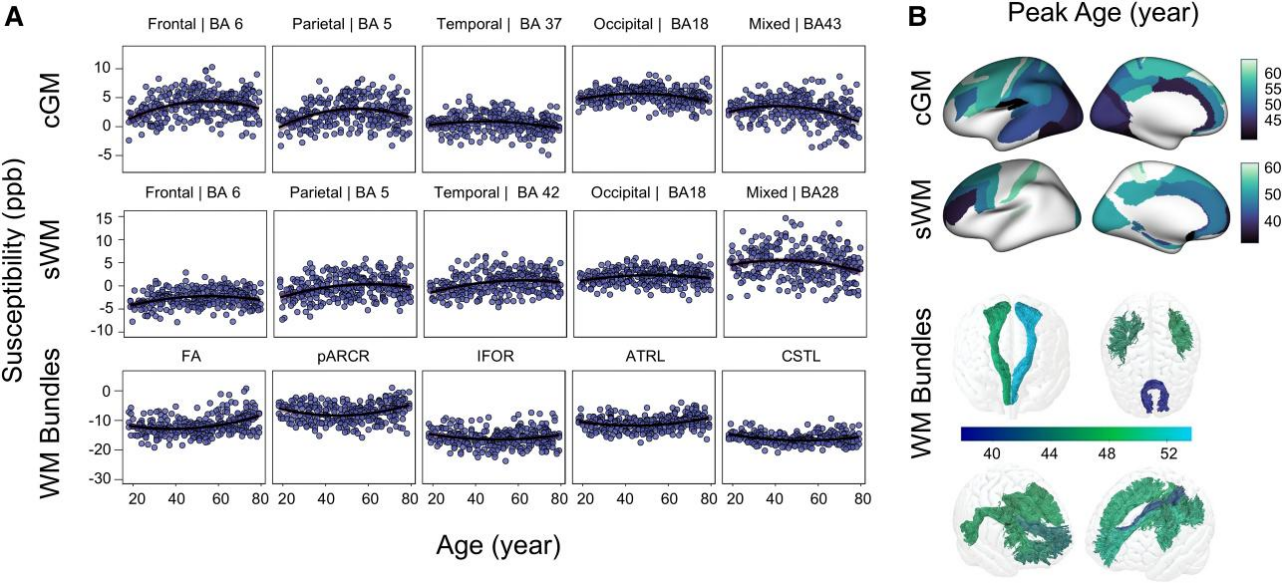
**Modelling QSM as a function of age. (A)** shows representative regions across five different lobes (frontal, parietal, temporal, occipital and mixed—shown from left to right) in the cGM (first row), sWM (second row) and WM bundles (third row). Compared to *R*_1_ and *R*_2_*, the susceptibility distribution is significantly noisier, with the most notable dispersion observed in the medial temporal areas, as exemplified by Brodmann area 28 in sWM. Scatter plots show individual participants (*N* = 293), with quadratic polynomial regression models (solid lines) fitted separately for each region. Shaded areas indicate 95% confidence intervals based on bootstrap resampling (*N* = 10 000). Model fits were compared to linear alternatives using likelihood-ratio tests. Linear and quadratic models were compared using likelihood-ratio tests, and quadratic fits were generally preferred (*P* > 0.05). The significance of the age and age^2^ effects was assessed using t-tests on the regression coefficients. Across regions, age^2^ terms were significant (*t* = −4.71–4.61; all *P* < 0.05), whereas age terms showed more variability (*t* = −4.45–5.15; *P* < 0.001–0.11); (**B)** the peak ages for the corresponding QSM quadratic age model, encoded on the inflated brain for cGM and sWM and in the respective WM bundles. Regions without significant quadratic fitting were removed. The colour bar represents the peak-age colour coding for each subregion. Displayed values reflect bilateral averages. QSM: quantitative susceptibility mapping, cGM: cortical grey matter, sWM: superficial white matter, WM: white matter, BA: Brodmann area.

QSM analysis revealed distinct age dependency patterns across different regions. (i) In cGM, most regions exhibited quadratic age dependencies with curves resembling those observed in *R*_1_ and *R*_2_*. However, certain areas (frontal area: BA 10, 11, 33, 47; parietal area: BA 3, 29; temporal area: BA 20, 21, 38; mixed transitional: BA 25, 26, 27, 28, 35, 36) showed only linear relationships with age in the likelihood-ratio test (*P* > 0.05). Due to the negative quadratic coefficient in the PR model, susceptibility decreases after reaching a maximum value. (ii) In sWM, the quadratic curves tended to be flatter or transition to linear in most regions, with the highest *R*^2^ of quadratic fitting observed in BA 10 at the frontal pole (*R*^2^ = 0.14, *P* < 0.001). (iii) In most WM bundles, the upward-opening curve in the quadratic model suggested that susceptibility values decrease first and then increase with age, in contrast to the patterns in cGM. The right superior longitudinal fasciculus showed the strongest quadratic age fitting across WM bundles (*R*^2^ = 0.16, *P* < 0.001) (see Fig. 4A).

For the covariates, our results showed that sex could be a significant factor for the qMRI age model especially in *R*_2_*, where we found significant sex effects in a substantial proportion of the brain regions (cGM:12/41, sWM:29/41, WM bundles:18/22), followed by QSM (cGM:7/41, sWM:13/41 and WM bundles:5/22), and *R*_1_ (cGM:4/41, sWM:7/41 and WM bundles:6/22).

For cognition, no significant associations were observed in the *R*_1_ models. In *R*_2_*, models showed significant effects in 12/41 sWM regions, while only BA26 in cGM and 10/22 WM bundles showed significance. In contrast, QSM models showed significant effects in 13/41 cGM regions, while no significant effects were observed in sWM regions, and only 5/22 WM bundles, reflecting the differential distribution and contributions of iron in cGM and myelin in sWM, as captured by QSM and *R*_2_*.

Education levels showed significant effects in a limited number of regional models: (i) for *R*_1_, in 1/41 cGM and 3/41 sWM regions (0/22 WM bundles); (ii) for *R*_2_*, in 3/41 cGM and 2/22 WM bundles (0/41 sWM); (iii) for QSM, in 2/41 cGM, 6/41 sWM and 2/22 WM bundles.

Details of the fitting of age-quadratic regression models are provided in Supplementary Table 2.

### Model evaluation

Model evaluation results suggested: (i) For *R*_1_ metrics, the differences between the models were small, with both showing stable fits and an EXPV ratio (B-spline/PR) close to 1: 0.98 (± 0.16) for cGM, 1.02 (± 0.16) for sWM and 1.01 (± 0.18) for WM bundles. (ii) For *R*_2_* and QSM models (particularly for QSM), more models with EXPV ratios significantly deviating from 1 were observed, suggesting more deviation from the expected trends in quadratic regression fitting, as reflected by an average ratio of 0.88 (± 0.24) for *R*_2_* in sWM regions and 0.86 (± 0.17) for QSM in sWM. Detailed model evaluation results were presented in Supplementary Table 3.

### Ageing patterns across structures

**Figure 5:**
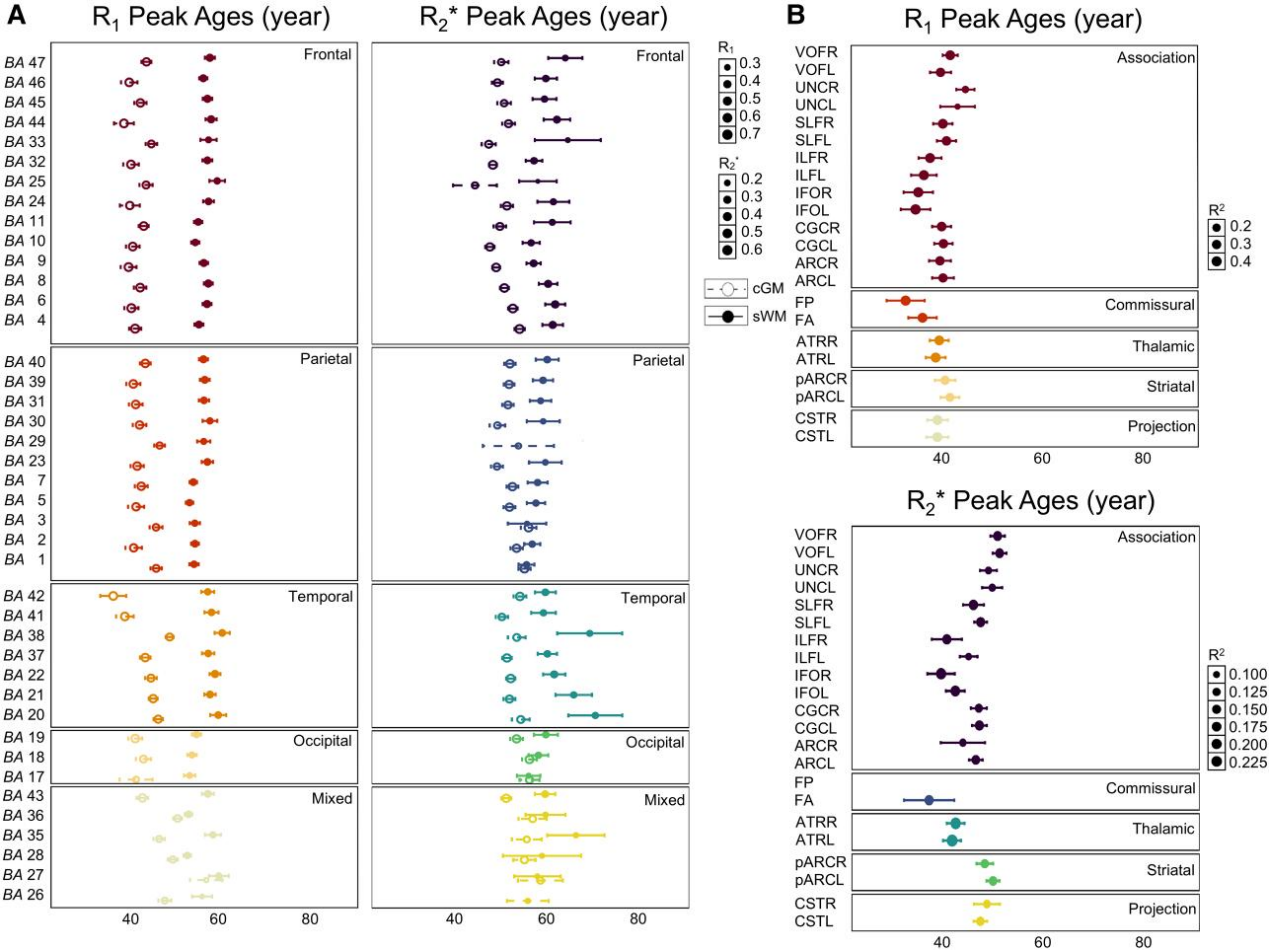
**Turning peak ages analysis. (A)** The peak ages determined by significant likelihood ratio tests from quadratic regression models (*P* < 0.05) for *R*_1_ and *R*_2_* metrics in cGM and sWM across BAs. Each data point represents the mean value of a specific BA, with an error bar showing SE derived from 10 000 bootstrap iterations. (**B)** Peak ages extracted among WM bundles in *R*_1_ and *R*_2_*. The data were organized based on the anatomical functional characteristics of different brain lobes or WM bundles. The experimental unit was the individual participant (*N* = 293). cGM: cortical grey matter, sWM: superficial white matter, BA: Brodmann area, FA: callosum forceps minor, FP: callosum forceps major, pARC: posterior arcuate fasciculus, ILF: inferior longitudinal fasciculus, SLF: superior longitudinal fasciculus, IFO: inferior fronto-occipital fasciculus, ARC: arcuate, ATR: thalamic radiation, CGC: cingulum cingulate, CST: corticospinal, L: left, R: right.

The analysis of peak ages using quadratic regression models for *R*_1_ and *R*_2_* metrics revealed a progression of turning points in age-related changes in tissue properties, shifting from deeper WM bundles to sWM and then to cGM: (i) *R*_1_ turning peaks occurred earliest in WM bundles, averaging at 39.16 ± 2.85 years, followed by sWM at 43.28 ± 3.71 years and latest in cGM, peaking at 56.40 ± 1.98 years (Figs 2B and 5A); (ii) *R*_2_* followed a similar pattern, with turning peaks at 45.06 years (± 5.43 years) in WM bundles, 52.23 ± 2.99 years in sWM and 60.27 ± 3.45 years in cGM (Figs 3B and 5A). (iii) In QSM analysis, however, some regional measurements failed in quadratic fitting with age, either showing significance only on the linear fitting or completely lacking age dependencies, as detailed in Supplementary Table 2. Regions with significant quadratic age fitting showed turning peaks at 46.81 years (±4.27 years) in WM bundles, 48.86 ± 7.38 years in sWM, and 51.54 ± 6.03 years in cGM (Fig. 4B).

The age-related models suggested that *R*_1_ peak ages exhibited lower variability across different brain structures than *R*_2_* (see Fig. 5). Nevertheless, they showed the most significant changes in the medial temporal regions and areas near the corpus callosum in mixed transitional areas, where these changes typically occur later. In contrast, *R*_2_* metrics displayed greater bootstrapping SEs in specific regions, such as the peak ages of BA 25 in both cGM and sWM, and BA 38 in sWM, indicating less stability in these regional measurements, or simply that the quadratic model was less justified in those regions.

### Age covariation analysis

**Figure 6:**
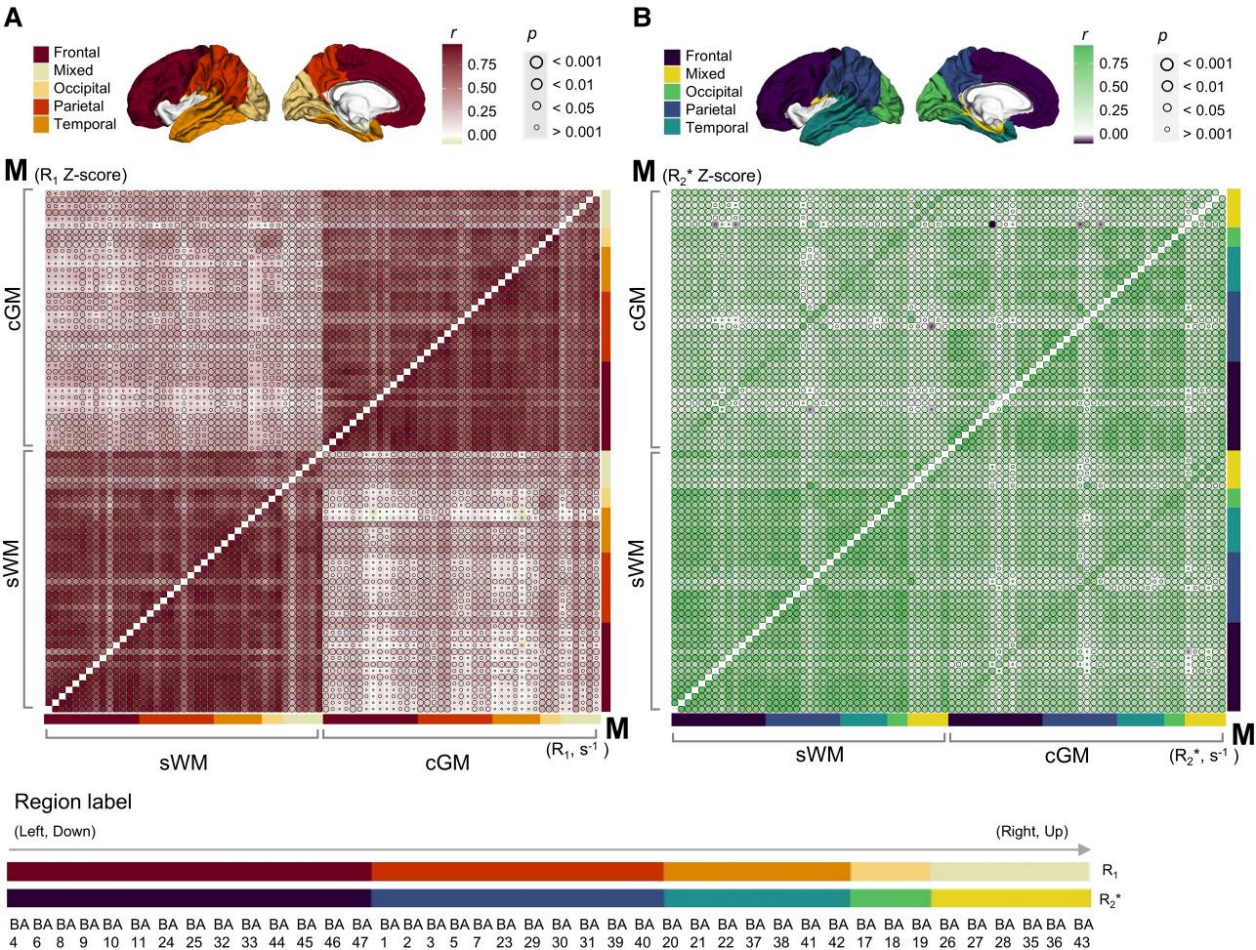
Correlation analysis of brain cGM and sWM. The figure presents Pearson correlation matrices for **(A)**  *R*_1_, and **(B)**  *R*_2_*, showing both intra- and inter-regional connectivity patterns within and between cGM and sWM. Within each matrix, the upper triangles depict z-scores derived from the residuals of significant quadratic models adjusted for ageing effects, together with sex, education and cognition as covariates, illustrating correlations that are independent of these factors. The lower triangles display the raw average values for each region, highlighting the overall correlations. The circle size increases as the *P*-value decreases, and the darker color show the increased correlation (as shown on the colourbar). Only regions with significant quadratic regression fits were included in the matrix calculations. Each submatrix (cGM and sWM) is subdivided into five lobes based on the anatomical locations of the selected BAs (coded as on the surface maps above the panel and the labels on the right-hand side of each panel). Region labels show the order and corresponding position of each brain region within the matrices. Pearson correlation coefficients (**r**) w**i**th corresponding *P*-values are shown. The experimental unit was the individual participant (*N* = 293). cGM: cortical grey matter, sWM: superficial white matter, BA: Brodmann area.

The intra-correlation (*R*_1_, *R*_2_* and susceptibility) within sWM and cGM BAs showed a general decrease accounting for age effects using the quadratic model (Fig. 6). Within cGM regions, the average correlation coefficient was 0.74 (qMRI) and 0.61 (ZqMRI) for *R*_1_, while for *R*_2_* it was 0.55 (qMRI) and 0.45 (ZqMRI). Within sWM regions, the average correlation coefficient was 0.74 (qMRI) and 0.68 (ZqMRI) for *R*_1_, while for *R*_2_* it was 0.63 (qMRI) and 0.58 (ZqMRI).

It should be highlighted that, as opposed to the intra-regional correlation, the removal of the modelled age dependence in the inter-regional correlation effectively increases the mean coefficient observed in *R*_1_ (0.26 for qMRI, 0.33 for ZqMRI) (Fig. 6A). The frontal, parietal and occipital lobes displayed high correlation coefficients in the intra-regional areas. In contrast, the temporal lobe and the medial temporal structures in the mixed category exhibited relatively minor correlation coefficients, both within themselves and with the remaining cortical regions. This could be attributed to the smaller size and lower signal-to-noise ratio. The auditory cortex's sWM (BA 41 and BA 42) showed the most significant change in correlations with the remaining sWM regions (high correlation coefficient and *P*-values) and with the remaining cGM regions (low correlation coefficient and *P*-values). While the frontal, parietal, and occipital lobes displayed strong correlations of values across intra-regions, the temporal lobe and the medial temporal structures (in the mixed category) showed smaller levels of correlation coefficients, both within themselves and to the remaining cortical regions.

Compared with *R*_1_, *R*_2_* showed an increased mean inter-regional correlation coefficient (0.71 for qMRI, 0.69 for ZqMRI) (Fig. 6B), as indicated in the diagonal entries of both the upper-left and lower-right sections. For *R*_2_*, those diagonal entries were comparable to, or larger than, the correlation coefficient across cGM or sWM BAs. Within the intra-correlational matrix for cGM and sWM, similar to *R*_1_, the lowest correlation coefficients were observed in BA 23, 26–29 and 33, 35, which correspond to splenial and entorhinal areas, rather than neocortical regions.

Given the reduced age dependence observed for QSM in most cGM and sWM regions (see in Supplementary Table 1), the expected outcome showed stable correlational matrices after accounting for age. Unlike the *R*_1_, *R*_2_* correlational matrices, where statistically significant correlations were consistently positive, most susceptibility correlations were positive for intra-connectivities within cGM and sWM, but negative for inter-connectivities. QSM covariation analysis was presented in Supplementary Fig. 1.

## Discussion

Using multiple qMRI mapping techniques, our results identified an ageing gradient based on microstructural properties across brain tissues, with quadratic model-based turning points of microstructural changes initially showing in deeper WM bundles during early adulthood, progressing to cortical-adjacent WM in middle adulthood, and culminating in the GM of cortical regions in late adulthood. This gradient was consistently observed across qMRI metrics (*R*_1_ and *R*_2_*), showing homogeneous ageing patterns mapped by different tissue properties. Beyond prior studies, our work offers several new perspectives: (i) comparison of age-related changes across regions and tissue types using qMRI, providing a multidimensional view of brain ageing; (ii) contrast of normative age models across qMRI metrics, showing distinct sensitivities to microstructural change with age; (iii) extension of qMRI analyses to cortical subdivisions, including primary and association cortices, with comparison to adjacent sWM and (iv) demonstration that qMRI-derived normative turning points differ from prior morphometric findings such as cortical thickness, yielding new insights that link microstructural and macroscopic perspectives in brain ageing.

### qMRI distribution

*R*_1_ relaxation rates vary between primary and associative cortical regions, similar to what has been previously reported for ‘Myelin-sensitive’ approaches.^65^ The distribution of *R*_2_* and susceptibility values across brain regions reflected their associations with iron and myelin content. Specifically, the increased *R*_1_, *R*_2_* and susceptibility values in the primary motor and visual cortices suggested substantial concentrations of both myelin and iron. We observed increased *R*_2_* values within the sWM in the primary and supplemental visual cortex, lateral frontal and temporal areas. In *R*_1_, there was a significant relative reduction in the primary visual cortex compared to other cortical regions. Both of these observations suggested that our measurements at 3 T were sensitive to iron-rich oligodendrocytes in sWM, similar to what had been previously demonstrated at 7 T.^66^  *R*_2_* and susceptibility maps in cGM showed a high correlation coefficient, as it has also been observed in both high-field cGM^27^ and in deep GM.^63^ Yet, in sWM, this behaviour changed substantially. While *R*_2_* values increased in sWM of the primary motor and supplemental motor regions, those regions showed below-average values in QSM. This observation suggested that *R*_2_* and susceptibility metrics might be dominated by the diamagnetic contribution of myelin in those regions.^67,68^ Finally, the diamagnetic dominance of myelin in WM was clear on the susceptibility quadratic fits of WM bundles, where a U-shape curve was observed (Fig. 4).

### Iron and myelin contributions

It is well-established that the brain lacks effective mechanisms for removing iron deposits,^69^ which are expected to accumulate progressively throughout adulthood.^2^ Our observation of later *R*_2_* peak ages in adulthood (as shown in Fig. 3A and B) can be explained by the increased contribution of iron with age, which shifts the peak ages associated with myelination. This interpretation is further supported by supplementary analyses of the present cohort (Supplementary Fig. 2), where *R*_1_, *R*_2_*, QSM quadratic fits of the ventral pallidum, a subcortical structure with high iron content, revealed later peak ages compared with cortical regions.

The average peak-age difference between *R*_1_ and *R*_2_* quadratic curves was largest in sWM (∼9 years), followed by WM bundles (∼6 years) and cGM (∼4 years), suggesting either an accelerated rate of iron deposition or reduced variation in myelination in these structures with age. *R*_2_* supported the observed ageing gradient (from WM to GM) but exhibited increased sensitivity to sex differences (compared with *R*_1_, QSM) in specific regions (see in Supplementary Table 2), which has not been reported in previous studies on *R*_2_* age modelling.^70,71^ Notably, the fitting age models showed that generally the peak ages of *R*_2_* happened later than those of *R*_1_ in the cortex (which should be attributed to the increased sensitivity to iron of *R*_2_*). QSM's performance was less consistent in modelling the age dependencies, with some regions displaying flat or nonsignificant trends, likely due to its higher susceptibility to artefacts close to the brain boundaries.^72^ This observation is significantly different from the strong increase in function of age seen in the basal ganglia regions.^44^ A confounding factor in the interpretation of QSM is that it reflects a mixture of diamagnetic and paramagnetic sources. One approach to addressing this is the use of χ-separation techniques, which disentangle diamagnetic and paramagnetic contributions.^73^ Previous studies have shown that χ-separation can measure more specific tissue markers and reduce QSM variability,^73,74^ but it should be noted that the most theoretically sound methods rely on the use of an *R*_2_ map (which was not available in this study).^74^ New AI-powered methods have started performing χ-separation fully based on *R*_2_* data^75^ and may contribute to (together with the *R*_1_ mapping) getting trajectories of those underlying mechanisms. Another avenue to isolate the contributions of iron and myelin would be to perform Macromolecular Tissue Volume Fraction (primarily driven by myelin in WM). Traditionally, this is accomplished using multi-echo variable flip-angle acquisitions,^41^ but this has recently been extended to using the MP2RAGE^76^ in combination with a dictionary matching approach to estimate *S*_0_ and *R*_1._^44^ Additionally, similar to previous reports showing QSM and *R*_2_* correlations with cognition in cGM,^71,77,78^ our model suggested that cognition shows significant correlations in both QSM and *R*_2_* models, while education had a smaller effect. However, cognition in the present study was assessed only with the MoCA, a coarse measure that spans multiple cognitive domains. To better interpret the impact of cognition on qMRI values and age modelling, more hypothesis-driven and domain-specific assessments would allow relating a specific cognitive aspect in a Brodmann area without overfitting or losing detection power.

### Quantitative MRI-based ageing patterns

There is substantial agreement between some of our findings and previous data available in the literature. Considerable evidence supports the pattern of *R*_1_ in WM development progressing from central-to-peripheral regions with earlier maturation in central compared to peripheral areas.^13^ In addition, it has been shown that *R*_1_ values for various WM tracts peak between 30 and 50 years,^41^ which aligns with our observation of the turning points of *R*_1_ in deeper WM tracts during early adulthood. Furthermore, *R*_1_ peaks around 40 years in WM bundles have been reported, along with the greater variability in *R*_2_*,^70^ which is consistent with our data. In contrast, the sWM, which lies just beneath the cortical mantle and consists mainly of slow-myelinating short-association fibres known as U-fibres, continues to develop into the fourth decade of life.^17^ Our findings confirm that sWM reaches its peak in microstructural properties in mid-adulthood, a pattern distinct from the earlier stabilization of WM bundles and the later development of cGM. The later peak ages in cGM may be due to iron deposition, water content and lipid composition, including contributions from both myelin bilayers and other cell membranes.^42,79,80^ Previous studies have shown that global cortical *T*_1_ values, which are inversely related to *R*_1_, decrease with age and are negatively correlated from the third to the eighth decade of life.^47^ These studies documented the turning point of *R*_1_ occurring between 40 and 60 years, aligning with our results, which show that *R*_1_ changes progress from sWM in mid-adulthood to cGM in late adulthood. These consistent observations across multiple studies support a sequential ageing gradient from deeper WM to cortical regions.

Our results are also aligned with existing research on lobe-specific cortical ageing patterns within the brain, as well as findings on WM tracts. In cGM, we observed that the temporal lobe, crucial for complex auditory processing, language and memory, matures later, in line with the extended developmental timeline into early adulthood previously reported.^81,82^ The frontal and parietal lobes (with roles in higher-order cognitive functions and sensory integration) showed intermediate peak ages in tissue properties, with the parietal structures reaching earlier than the frontal ones, corroborating previous findings.^23^ It is interesting to note that cGM, sWM, as well as WM bundles associated with the motor cortex, had the earliest peak ages derived from *R*_1_ (Fig. 5), reflecting rapid motor processing development (and earlier decline). This finding aligned with the earlier turning peaks observed in the motor-related CST bundle, which occurred at ∼39 years of age in *R*_1_ model (Supplementary Table 2), and in the visual-related FP, which reached its turning peak earlier than CST at 32.77 years old (both showing peak ages earlier than most bundles). This supports the inferior-to-superior developmental theory, which posits that earlier age-related development facilitates motor functions and visual areas. Within *R*_1_, the next earlier peak was observed in the FA (36.14 years), which connected prefrontal cortices involved in higher-level motor control, and the IFO (around 34 years), implicated in visual processing, reading and semantic processing. In contrast, temporal lobe regions supporting higher-order cognitive processes matured later, with other associative fibres maturing in the early fourth decade. This gradient demonstrates that basic sensory and motor regions mature earlier, supporting both inferior-to-superior and posterior-to-anterior brain development models, and provides a sequential understanding of specific regional brain ageing patterns.^19,41,83^

Compared to previous research, this qMRI study provides a comprehensive and novel research framework beyond traditional morphology. One important aspect is the observation that the peak ages derived from quadratic fits in cGM (∼60 years old) are significantly later than those estimated using cortical thickness,^5,6,9^ showing that thickness and qMRI present different aspects of brain ageing. This observation aligns with an earlier finding that the volumetry of basal ganglia nuclei is independent of the derived relaxation and susceptibility measurements.^44^ In Supplementary Fig. 3, we showed that cortical thickness in this same cohort exhibits distinct trajectories compared to those derived from qMRI parameters. This difference suggested that cortical thickness and qMRI metrics capture different biological aspects of brain ageing in adulthood, with the former reflecting macroscopic atrophy, which may be driven by dendritic and synaptic pruning or loss, and the latter being sensitive to microstructural tissue properties such as iron accumulation, water mobility, macromolecular pool and even vascular ageing.^26,42,80,84^

### Age covariation

In the covariation analysis, the greater reduction of the inter-regional correlation in *R*_1_ for cGM (0.13) versus sWM (0.06) could be expected from Fig. 2A, which shows a significant change as a function of age in cGM regions (both due to reduced variance and a polynomial curve that is more asymmetric with respect to the age range). *R*_2_* exhibited significant inter-regional correlations between sWM and cGM in the same BAs, which were not detected in *R*_1_, showing that iron deposition follows a similar pattern in adjacent sWM and cGM regions with a co-occurring increase or decrease of iron deposition. The differing correlational patterns observed in *R*_2_* and susceptibility metrics highlight their distinct tissue sensitivities. *R*_2_* (as *R*_1_) predominantly showed positive correlations, while QSM exhibited primarily negative correlations between sWM and cGM regions. This can be attributed to QSM being primarily influenced by the diamagnetic effects of myelin in sWM,^85^ whereas in cGM it is predominantly affected by the paramagnetic effects of iron concentration.^86^ Notably, these two quantities were generally correlated.

### Limitation

This study has several limitations; one concerns the accuracy of the applied BA parcellation. Although the intermediate level of granularity helps balance sensitivity and interpretability, reducing the risk of noise associated with over-fine parcellations, it does not fully align with functional myeloarchitectural distributions.^65^ A function-based atlas may therefore be more suitable when the objective is to examine the relationship between specific cognitive abilities (e.g. visual acuity, reaction time and language fluency) and qMRI metrics. Additionally, while changes in myelination and iron deposition are generally considered primary contributors to age-related variations in qMRI metrics, other factors may also be involved. For instance, non-myelin membranes (microtubules perpendicular to the axonal axis^87-89^) and vascular ageing processes,^84^ such as increasing arterial stiffness,^90^ could exert additional influences on parameters including relaxation rates and magnetic susceptibility. These aspects should therefore be regarded as additional considerations when interpreting the present findings.

Another limitation of this study concerns the referencing of QSM values. In this study, the whole-brain mean was used as the reference, which is one of the consensus recommendations.^91^ Previous work has shown that commonly used reference regions, such as cerebrospinal fluid (CSF), exhibit minimal inter-individual variability and small dependence on age^2^. Our approach ensures comparability across participants by avoiding potential variance from flow artefacts in the CSF QSM values. However, global susceptibility shifts, such as age-related increases in deep GM susceptibility or changes in WM/GM volume ratios, could bias age modelling and induce spurious correlations between QSM values across regions.^92^

Moreover, our study did not correct for axonal^37,38^ or cortical surface^93^ orientation relative to the static magnetic field (*B*_0_). Although such effects are less pronounced at 3T, they may still influence *R*_2_*. In sWM and cGM, orientation effects are likely to average out across subjects due to cortical folding. Nonetheless, we cannot entirely rule out their contribution to the observed cGM–sWM correlations, even if axon orientation is not the primary driving factor. In WM bundles aligned with *B*₀, such as the CST tract, myelin sensitivity may be reduced compared to more perpendicularly oriented bundles like the forceps major or minor. However, orientation within each ROI is consistent across subjects and thus unlikely to bias the estimated peak ages.

Additionally, qMRI metrics in WM bundles were analysed separately for each hemisphere, supported by the evidence of distinct ageing trajectories in the left and right hemispheres^70^ with the right bundles systematically peaking earlier in *R*_1_. In contrast, we have an averaging of measurements across hemispheres for cortical areas, although following the empirical approach of most current studies and based on evidence indicating minimal hemispheric differences in morphometrics and quantitative measurements.^94,95^ This may overlook subtle lateralization effects. Beyond hemispheric considerations, another limitation is that our analyses did not include subcortical regions, as this study primarily focused on regional ageing patterns from cortex to WM. Nevertheless, incorporating analyses of deep GM given their established role in age-related iron accumulation and the greater robustness of QSM quantification in basal ganglia and deep GM structures, could have offered a better handle on the interaction between iron concentration and the shift of peak ages.^44,72^

## Conclusion

Overall, brain microstructural ageing patterns derived from qMRI offer a novel perspective for studying both healthy and pathological ageing, enabling the differentiation of brain region and tissue-specific ageing patterns. Our study therefore provides essential grounds for future MRI-based ageing studies.

## Supplementary Material

fcag010_Supplementary_Data

## Data Availability

The original MR data, edited scripts and related materials have been shared on public data repositories and GitHub (https://doi.org/10.34973/7q0a-vj19, https://doi.org/10.34973/v364-0d37, https://github.com/JosePMarques). Details on prior use of the published cohort are provided in Supplementary Method 2. Additional materials can be obtained from the corresponding author upon reasonable request (jose.marques@donders.ru.nl).
