# Supplementary material for: Mapping heterogeneous region- and tissue-specific brain ageing patterns using quantitative MRI
Source: Brain Commun. 2026 Jan 13;8(1):fcag010. doi: 10.1093/braincomms/fcag010 (PMC12895350; doi:10.1093/braincomms/fcag010)
Supplement: fcag010_Supplementary_Data [file fcag010_supplementary_data.pdf]

Supplementary Materials **Page**

Supplementary Materials	Page
Supplementary Method 1: Descriptions of MRI Data Processing	1-4
Supplementary Method 2: Prior Use of the ABRIM Cohort	5-6
Supplementary Table 1: Quantitative Distibution	7-12
Supplementary Table 2: Polynomial Regression Analysis	13-18
Supplementary Table 3: Model evaluation analysis	19-24
Supplementary Figure 1: Quantitative Correlation Analysis in QSM	25
Supplementary Figure 2: Normative Age Modelling in the Ventral Pallidum	26
Supplementary Figure 3: Normative Age Modelling of Cortical Thickness	27

Supplementary Method 1: Descriptions of MRI Data Processing

1. Image Reconstruction

Preprocessing steps were conducted separately for the MP2RAGE and ME-GRE sequences to generate R_1, R_2*, and susceptibility maps. For the MP2RAGE data to create the T_1 maps, preprocessing involved transmit field inhomogeneity correction (B_1+ correction) to address variations in the radiofrequency field [1]. From the T_1 maps, R_1 maps were calculated (R_1=1/T_1) and these values will be discussed throughout this work. Background noise removal of the MP2RAGE data was performed using in-house scripts [2].

For the ME-GRE data, preprocessing included QSM phase unwrapping and background field removal, followed by the reconstruction of R_2* and susceptibility maps using the SEPIA toolbox (version 1.2.2.4) [3]. The pipeline used has been extensively described in a publication using Site 1 data [4] and relies on the following methods: ROMEO for field calculation [5]; V-SHARP for background field removal [6]; LPCNN for dipole inversion [7]; and the ARLO technique for R_2* maps computation [8].

R_2* and susceptibility maps were co-registered to the R_1 (MP2RAGE) space. A rigid body transformation matrix between the first echo of the ME-GRE sequence and the second inversion time image from the MP2RAGE sequence was obtained using FSL [9] and was subsequently applied to bring the R_2* and susceptibility maps to the R_1 map space.

Diffusion-weighted images (DWIs) were preprocessed using the default settings of QSIprep (version 0.18.0) [10], which includes denoising and motion correction.

2. Cortical Parcellation

Cortical segmentation was conducted using FreeSurfer (version 6.0) [11] with MP2RAGE images. In this step, the recon-all pipeline was applied to the MP2RAGE data, following the methodology outlined in Fujimoto et al.,[12] where the UNI image was first multiplied by the INV2 image to reduce background noise. T1-weighted images from patients were lesion-

filled by FSL earlier. Cortical parcellation was performed using the PALS-B12 Atlas [13] provided by FreeSurfer, with further in-house refinements to correct inaccurate labels. Manual quality control steps, including skull-stripping correction, pial surface error correction, and intensity normalisation, were implemented before and after segmentation to ensure the accuracy of the parcellation and the extraction of quantitative measurements. Manual corrections were required only when the automated segmentation failed or produced substantial errors (21 subjects, 7.2%)

3. White Matter Tractography

To obtain bundle information, a pyAFQ [14] pipeline of QSIprep was applied. This pipeline utilises multi-shell and multi-tissue spherical deconvolution methods to estimate fibre orientations and extract a specific set of 22 bundles. To calculate qMRI measurements for each WM bundle, fibre tracts were co-registered to R_1 (MP2RAGE) space (where R_2* and QSM had also been co-registered) using the transformation matrix computed from the flirt [9] co-registration of DWI data (brain-masked and distortion-corrected images) with the brain-masked second inversion time images of the MP2RAGE. The SCILPY toolbox [15] was used to extract median qMRI values in each fibre tract. To ensure that the measurements were focused on the core WM tissue and devoid of GM partial volume effects (PVE), only the central 70% of each fibre tract was retained (excluding the distal 15% at each end). For R_2*, head positions of subjects were controlled within ±10 degrees across the sample, which likely limits the influence of fibre orientation on data variability.

4. Quantitative Mapping Across Brain Structures

Quantitative surface maps were created by projecting qMRI maps onto the brain surface derived from FreeSurfer. qMRI values in the cortex were extracted in the middle cortical layer between the white and pial surfaces using FreeSurfer with settings 0.5 and -0.5, respectively[11]. The average qMRI measurements (R_1, R_2* and susceptibility values) derived from the individual medians were calculated separately for each region and hemisphere, considering only vertices that passed automatic quality assurance per region as described in a previous study [16]. In this step, outliers beyond three times the median were excluded before

calculating regional averages based on brain parcellation to account for potential vessel artefacts or segmentation errors.

5. Reference

1. Marques JP, Gruetter R. New developments and applications of the MP2RAGE sequence-focusing the contrast and high spatial resolution R1 mapping. *PloS One*. 2013;8(7):e69294.

2. O'Brien KR, Kober T, Hagmann P, et al. Robust T1-weighted structural brain imaging and morphometry at 7T using MP2RAGE. *PloS One*. 2014;9(6):e99676.

3. Chan KS, Marques JP. SEPIA—Susceptibility mapping pipeline tool for phase images. *Neuroimage*. 2021;227:117611.

4. Jansen MG, Zwiers MP, Marques JP, et al. The Advanced BRain Imaging on ageing and Memory (ABRIM) data collection: Study design, data processing, and rationale. *PloS One*. 2024;19(6):e0306006. doi:10.1371/journal.pone.0306006

5. Dymerska B, Eckstein K, Bachrata B, et al. Phase unwrapping with a rapid opensource minimum spanning tree algorithm (ROMEO). *Magn Reson Med*. 2021;85(4):2294-2308. doi:10.1002/mrm.28563

6. Li W, Wu B, Liu C. Quantitative susceptibility mapping of human brain reflects spatial variation in tissue composition. *NeuroImage*. 2011;55(4):1645-1656. doi:10.1016/j.neuroimage.2010.11.088

7. Lai KW, Aggarwal M, Van Zijl P, Li X, Sulam J. Learned Proximal Networks for Quantitative Susceptibility Mapping. In: Martel AL, Abolmaesumi P, Stoyanov D, et al., eds. *Medical Image Computing and Computer Assisted Intervention – MICCAI 2020*. Vol 12262. Springer International Publishing; 2020:125-135.

8. Pei M, Nguyen TD, Thimmappa ND, et al. Algorithm for fast monoexponential fitting based on Auto-Regression on Linear Operations (ARLO) of data. *Magn Reson Med*. 2015;73(2):843-850. doi:10.1002/mrm.25137

9. Jenkinson M, Beckmann CF, Behrens TEJ, Woolrich MW, Smith SM. FSL. *NeuroImage*. 2012;62(2):782-790. doi:10.1016/j.neuroimage.2011.09.015

10. Cieslak M, Cook PA, He X, et al. QSIPrep: an integrative platform for preprocessing and reconstructing diffusion MRI data. *Nat Methods*. 2021;18(7):775-778.

11. Fischl B. FreeSurfer. *Neuroimage*. 2012;62(2):774-781.

12. Fujimoto K, Polimeni JR, Van Der Kouwe AJ, et al. Quantitative comparison of cortical surface reconstructions from MP2RAGE and multi-echo MPRAGE data at 3 and 7 T. *Neuroimage*. 2014;90:60-73.

13. Van Essen DC. A Population-Average, Landmark- and Surface-based (PALS) atlas of human cerebral cortex. *NeuroImage*. 2005;28(3):635-662.

14. Kruper J, Yeatman JD, Richie-Halford A, et al. Evaluating the Reliability of Human Brain White Matter Tractometry. *Aperture Neuro*. 2021;2021(1):25. doi:10.52294/e6198273-b8e3-4b63-babb-6e6b0da10669

15. GitHub - scilus/scilpy: The Sherbrooke Connectivity Imaging Lab (SCIL) Python dMRI processing toolbox.

16. Shams Z, Norris DG, Marques JP. A comparison of in vivo MRI based cortical myelin mapping using T1w/T2w and R1 mapping at 3T. *PloS One*. 2019;14(7):e0218089.

Supplementary Method 2: Prior Use of the ABRIM Cohort

1. Previously published work by the authors using ABRIM data

Three previously published studies by members of the current authors have used data from the ABRIM cohort:

- ABRIM cohort description: This publication provides an overview of the cohort's design, recruitment, MRI protocol, and available neuropsychological measures [1].
- Quantitative MRI (qMRI) normative study [2]: This study modelled normative trajectories of R_1, R_2*, and magnetic susceptibility in the basal ganglia.
- qMRI-based brain-age study [3]: This study included ABRIM healthy control qMRI data as part of a combined cohort sample to train a brain age predictive model.

2. Data overlaps with the current study

- The present study and the ABRIM cohort description paper [1] both rely on the same participant pool and shared MRI/behavioural measures. However, the previous publication did not include any imaging analyses relevant to the current study, including brain segmentation, cortical parcellation, image co-registration, computation of regional R_1, R_2*, and magnetic susceptibility values, or normative age modelling.
- The deep grey matter-based qMRI normative study [2] also used data from the ABRIM cohort, relying on the same underlying participant pool but with a smaller subset (260 participants versus 293 in the present study). The two studies target different regions of interest: Chan et al. focused exclusively on deep grey matter structures (basal ganglia), whereas the present study investigates cortical grey matter, superficial white matter, and deep white matter regions. In addition to the different anatomical focus, the normative age-modelling approaches also differ: the previous study used general linear models and Gaussian process regression to model age trajectories in deep gray matter, whereas the present study employs polynomial regression and a more flexible cubic B-spline model tailored to cortical and white-matter regional analyses. The qMRI normative analysed in the present study, therefore, does not overlap with that examined in the study, and the analyses address entirely distinct research questions.
- The brain-age predictive study [3] also included data from the ABRIM cohort, but only as one component of a combined cohort sample used for brain-predicted age modelling. The published research combined quantitative MRI measures across

different cohorts and harmonized cross-cohort data, whereas the present study focuses exclusively on the ABRIM dataset. In addition, the previous study did not perform any normative age modelling; instead, it focused on estimating brain-predicted age differences using machine-learning models. The brain segmentation, cortical parcellation, and computed regional R_1, R_2*, and magnetic susceptibility values differ entirely between the two studies. The qMRI metrics and regional analyses conducted in the present study, therefore, do not overlap with those used in the brain-age analysis, and the two studies address distinct research questions.

- No other published work by the authors has used this cohort. External researchers may have used the publicly available ABRIM dataset. But these works do not involve any of the authors of the present manuscript, are unrelated to the present study's research aims, and did not involve qMRI-based normative analysis.

3. Reference

1. Jansen MG, Zwiers MP, Marques JP, et al. The Advanced BRain Imaging on ageing and Memory (ABRIM) data collection: Study design, data processing, and rationale. *PLoS One*. 2024;19(6):e0306006. doi:10.1371/journal.pone.0306006

2. Chan KS, Zwiers MP, Jansen MG, et al. Normative trajectories of R1, R2* and magnetic susceptibility in basal ganglia on healthy ageing. *Imaging Neuroscience*. Published online 2024. Accessed January 10, 2025. https://direct.mit.edu/imag/article/doi/10.1162/imag_a_00456/127408

3. Chen X, Lu PJ, Ocampo-Pineda M, et al. Unraveling Microstructural and Macrostructural Brain Age Dynamics in Multiple Sclerosis. *Neurol Neuroimmunol Neuroinflamm*. 2025;12(5):e200459. doi:10.1212/NXI.0000000000200459

Supplementary Table 1: Quantitative Distibution

Region	Mean	SD
**R1-cGM (s-1)**		
**Frontal Lobe**		
BA4	0.75	0.02
BA6	0.71	0.02
BA8	0.70	0.02
BA9	0.69	0.01
BA10	0.70	0.01
BA11	0.69	0.01
BA24	0.67	0.01
BA25	0.64	0.01
BA32	0.68	0.01
BA33	0.65	0.01
BA44	0.70	0.01
BA45	0.71	0.01
BA46	0.70	0.01
BA47	0.70	0.01
**Parietal Lobe**		
BA1	0.75	0.02
BA2	0.71	0.01
BA3	0.74	0.02
BA5	0.71	0.01
BA7	0.71	0.01
BA23	0.68	0.01
BA29	0.68	0.01
BA30	0.68	0.01
BA31	0.69	0.01
BA39	0.70	0.01
BA40	0.70	0.01
**Temporal Lobe**		
BA20	0.66	0.01
BA21	0.67	0.01
BA22	0.69	0.01
BA37	0.69	0.01
BA38	0.66	0.01
BA41	0.70	0.01
BA42	0.72	0.02
**Occipital Lobe**		
BA17	0.73	0.01
BA18	0.72	0.01
BA19	0.71	0.01
**Mixed**		
BA26	0.68	0.02
BA27	0.69	0.01
BA28	0.69	0.02
BA35	0.67	0.01
BA36	0.68	0.01
BA43	0.70	0.01
**R1-sWM (s-1)**		
**Frontal Lobe**		
BA4	1.07	0.04
BA6	1.07	0.04
BA8	1.06	0.04
BA9	1.06	0.04
BA10	1.06	0.04
BA11	1.05	0.04
BA24	1.07	0.04
BA25	0.97	0.05
BA32	1.08	0.04
BA33	1.03	0.05
BA44	1.08	0.04
BA45	1.05	0.04
BA46	1.07	0.04
BA47	1.05	0.04

**Parietal Lobe**		
BA1	1.04	0.03
BA2	1.07	0.04
BA3	1.05	0.03
BA5	1.03	0.04
BA7	1.05	0.04
BA23	1.06	0.04
BA29	1.00	0.05
BA30	1.06	0.04
BA31	1.07	0.04
BA39	1.07	0.04
BA40	1.04	0.04
**Temporal Lobe**		
BA20	1.02	0.04
BA21	1.05	0.04
BA22	1.09	0.04
BA37	1.07	0.04
BA38	0.98	0.04
BA41	1.09	0.04
BA42	1.09	0.04
**Occipital Lobe**		
BA17	1.00	0.03
BA18	1.04	0.03
BA19	1.07	0.04
**Mixed**		
BA26	1.01	0.05
BA27	0.97	0.05
BA28	1.02	0.03
BA35	1.03	0.03
BA36	0.96	0.03
BA43	1.06	0.04
**R1-WM bundles (s-1)**		
ARCL	1.16	0.04
ARCR	1.17	0.04
ATRL	1.16	0.05
ATRR	1.17	0.05
CGCL	1.14	0.04
CGCR	1.14	0.04
CSTL	1.13	0.04
CSTR	1.14	0.04
FA	1.19	0.05
FP	1.14	0.05
IFOL	1.15	0.05
IFOR	1.15	0.05
ILFL	1.14	0.04
ILFR	1.16	0.04
pARCL	1.15	0.04
pARCR	1.16	0.04
SLFL	1.15	0.04
SLFR	1.17	0.04
VOFR	1.15	0.04
VOFL	1.15	0.04
UNCR	1.03	0.06
UNCL	1.02	0.06
**R2*-cGM (s-1)**		
**Frontal Lobe**		
BA4	19.24	1.05
BA6	17.57	0.88
BA8	16.82	0.82
BA9	16.56	0.76
BA10	17.13	0.84
BA11	17.74	1.12
BA24	16.02	1.00
BA25	16.26	1.66
BA32	16.40	0.88

BA33	15.04	1.06
BA44	17.75	0.91
BA45	17.71	0.96
BA46	17.50	0.90
BA47	17.67	1.14
**Parietal Lobe**		
BA1	18.37	0.88
BA2	18.06	0.87
BA3	18.15	0.82
BA5	17.19	0.79
BA7	18.07	0.82
BA23	17.62	1.23
BA29	17.79	1.59
BA30	17.49	1.07
BA31	17.74	1.09
BA39	18.10	0.95
BA40	17.51	0.85
**Temporal Lobe**		
BA20	16.76	1.19
BA21	17.03	1.15
BA22	17.69	0.92
BA37	18.32	1.00
BA38	15.51	1.11
BA41	17.95	0.98
BA42	18.56	1.07
**Occipital Lobe**		
BA17	19.83	1.02
BA18	19.96	0.95
BA19	19.08	0.88
**Mixed**		
BA26	18.99	1.86
BA27	17.71	1.38
BA28	16.63	1.32
BA35	18.08	1.46
BA36	16.86	1.27
BA43	17.59	0.89
**R2*-sWM (s-1)**		
**Frontal Lobe**		
BA4	20.29	0.93
BA6	19.70	0.83
BA8	19.31	0.90
BA9	19.44	0.88
BA10	20.08	0.93
BA11	20.12	1.04
BA24	19.68	0.98
BA25	18.72	1.54
BA32	19.80	0.96
BA33	18.70	1.14
BA44	20.66	0.91
BA45	20.47	1.07
BA46	20.51	0.92
BA47	20.51	1.23
**Parietal Lobe**		
BA1	20.33	0.90
BA2	20.24	0.88
BA3	20.39	0.83
BA5	19.47	0.88
BA7	20.26	0.89
BA23	20.20	1.22
BA29	19.11	1.35
BA30	19.70	1.09
BA31	20.42	1.07
BA39	20.70	0.93
BA40	20.15	0.90
**Temporal Lobe**		

BA20	19.49	1.08
BA21	20.33	1.15
BA22	20.97	0.94
BA37	20.79	0.98
BA38	18.88	1.17
BA41	20.26	0.97
BA42	20.80	0.99
**Occipital Lobe**		
BA17	20.64	0.99
BA18	21.13	0.94
BA19	20.92	0.91
**Mixed**		
BA26	19.33	1.46
BA27	19.31	1.42
BA28	19.24	1.31
BA35	19.80	1.36
BA36	18.71	1.26
BA43	20.19	0.97
**R2*-WM bundles (s-1)**		
ARCL	21.05	1.08
ARCR	20.65	1.03
ATRL	21.20	1.18
ATRR	20.96	1.21
CGCL	20.51	1.09
CGCR	20.43	1.11
CSTL	19.65	1.03
CSTR	19.75	1.02
FA	21.21	1.21
FP	21.61	1.40
IFOL	21.24	1.12
IFOR	21.21	1.14
ILFL	20.88	1.15
ILFR	21.01	1.13
pARCL	20.39	1.04
pARCR	20.35	1.09
SLFL	20.98	1.02
SLFR	21.09	0.98
VOFR	20.71	1.10
VOFL	20.55	1.02
UNCR	19.72	1.14
UNCL	19.66	1.24
**Susceptibility-cGM (ppb)**		
**Frontal Lobe**		
BA4	8.37	2.33
BA6	3.70	2.26
BA8	0.97	1.97
BA9	0.13	1.73
BA10	0.24	1.49
BA11	-1.34	0.98
BA24	1.09	2.10
BA25	-2.50	2.67
BA32	2.88	2.11
BA33	1.26	2.81
BA44	4.73	2.57
BA45	3.29	2.01
BA46	0.97	1.80
BA47	0.66	1.96
**Parietal Lobe**		
BA1	2.34	1.40
BA2	4.70	2.03
BA3	4.76	2.34
BA5	2.35	2.29
BA7	2.44	1.72
BA23	7.75	2.91
BA29	0.50	4.90

BA30	4.01	2.71
BA31	6.65	2.45
BA39	3.81	1.86
BA40	2.74	1.59
**Temporal Lobe**		
BA20	-1.98	1.23
BA21	-1.22	1.52
BA22	1.58	1.79
BA37	0.52	1.56
BA38	-3.99	1.73
BA41	3.96	2.86
BA42	8.34	2.46
**Occipital Lobe**		
BA17	6.11	1.69
BA18	5.27	1.34
BA19	4.73	1.66
**Mixed**		
BA26	-1.07	6.09
BA27	2.65	2.83
BA28	2.75	2.77
BA35	0.88	2.97
BA36	0.62	1.94
BA43	2.76	2.23
**Susceptibility-sWM (ppb)**		
**Frontal Lobe**		
BA4	-5.59	2.42
BA6	-2.75	1.84
BA8	-0.17	1.87
BA9	2.00	1.84
BA10	4.84	2.13
BA11	0.34	1.42
BA24	5.99	2.88
BA25	-0.33	2.61
BA32	4.77	2.57
BA33	7.73	3.16
BA44	2.06	2.21
BA45	3.57	2.27
BA46	3.86	2.07
BA47	3.52	2.66
**Parietal Lobe**		
BA1	-2.73	1.92
BA2	-1.15	1.97
BA3	-2.80	1.80
BA5	-0.30	2.48
BA7	0.03	1.98
BA23	6.88	3.63
BA29	3.69	5.23
BA30	2.86	3.65
BA31	5.50	3.09
BA39	2.96	2.23
BA40	1.80	2.02
**Temporal Lobe**		
BA20	-0.26	1.57
BA21	1.85	1.74
BA22	3.62	2.25
BA37	1.40	1.84
BA38	1.41	1.97
BA41	-0.73	2.18
BA42	0.56	2.33
**Occipital Lobe**		
BA17	2.00	1.94
BA18	1.94	1.61
BA19	0.97	1.87
**Mixed**		
BA26	3.72	6.17

BA27	6.83	4.30
BA28	4.79	3.41
BA35	5.37	4.41
BA36	3.36	2.82
BA43	2.20	2.52
**Susceptibility-WM bundles (ppb)**		
ARCL	-9.21	2.02
ARCR	-10.45	2.33
ATRL	-11.20	2.39
ATRR	-11.64	2.38
CGCL	-5.29	2.75
CGCR	-4.97	2.87
CSTL	-16.43	1.86
CSTR	-17.37	2.10
FA	-11.85	3.29
FP	-21.23	3.32
IFOL	-16.76	2.32
IFOR	-16.12	2.94
ILFL	-14.20	2.66
ILFR	-13.82	3.23
pARCL	-7.36	3.00
pARCR	-7.51	3.21
SLFL	-9.51	2.01
SLFR	-9.79	2.16
VOFR	-8.84	3.20
VOFL	-9.69	3.25
UNCR	-8.20	2.65
UNCL	-7.66	3.06

Abbreviation: SD: standard deviation; cGM: cortical grey matter; sWM: superficial white matter; WM: white matter; FA: Callosum Forceps Minor; FP: Callosum Forceps Major; ARC: Arcuate; pARC: Posterior Arcuate Fasciculus; ATR: Thalamic Radiation; CGC: Cingulum Cingulate; CST: Corticospinal; IFO: Inferior Fronto-Occipital Fasciculus; ILF: Inferior Longitudinal Fasciculus; SLF: Superior Longitudinal Fasciculus; VOF: Vertical Occipital Fasciculus; UNC: Uncinate; L: left; R: right; BA: Brodmann Area.

Supplementary Table 2: Polynomial Regression Analysis

Region	β Age	p Age	β Age^2	p Age^2	β Sex	p Sex	p Low Education	p High Education	p MoCA	R^2	Peak Age	Peak Age (SE)
**R1-cGM**												
**Frontal Lobe**												
BA4	4.2e-03	p<0.0001	-3.8e-05	p<0.0001	-3.77E-04	p>0.05	p>0.05	p>0.05	p>0.05	0.41	55.30	1.00
BA6	3.4e-03	p<0.0001	-3e-05	p<0.0001	5.90e-04	p>0.05	p>0.05	p>0.05	p>0.05	0.48	57.13	1.00
BA8	3.3e-03	p<0.0001	-2.9e-05	p<0.0001	4.09e-04	p>0.05	p>0.05	p>0.05	p>0.05	0.49	57.42	1.00
BA9	2.9e-03	p<0.0001	-2.6e-05	p<0.0001	4.90e-04	p>0.05	p>0.05	p>0.05	p>0.05	0.44	56.41	0.95
BA10	2.9e-03	p<0.0001	-2.6e-05	p<0.0001	9.18e-04	p>0.05	p>0.05	p>0.05	p>0.05	0.34	54.48	0.92
BA11	2.7e-03	p<0.0001	-2.5e-05	p<0.0001	2.22e-03	p>0.05	p>0.05	p>0.05	p>0.05	0.37	55.14	0.93
BA24	2.1e-03	p<0.0001	-1.8e-05	p<0.0001	3.92e-04	p>0.05	p>0.05	p>0.05	p>0.05	0.37	57.48	1.21
BA25	1.7e-03	p<0.0001	-1.4e-05	p<0.0001	2.64e-03	p<0.05	p>0.05	p>0.05	p>0.05	0.31	59.42	1.75
BA32	2.5e-03	p<0.0001	-2.2e-05	p<0.0001	5.84e-04	p>0.05	p>0.05	p>0.05	p>0.05	0.40	57.22	1.11
BA33	1.7e-03	p<0.0001	-1.5e-05	p<0.0001	1.02e-03	p>0.05	p>0.05	p>0.05	p>0.05	0.25	57.48	1.82
BA44	2.9e-03	p<0.0001	-2.5e-05	p<0.0001	8.58e-04	p>0.05	p>0.05	p>0.05	p>0.05	0.48	58.06	1.24
BA45	3.1e-03	p<0.0001	-2.7e-05	p<0.0001	1.18e-04	p>0.05	p>0.05	p>0.05	p>0.05	0.47	57.23	1.06
BA46	3e-03	p<0.0001	-2.6e-05	p<0.0001	6.87e-04	p>0.05	p>0.05	p>0.05	p>0.05	0.44	56.30	0.91
BA47	3.1e-03	p<0.0001	-2.7e-05	p<0.0001	1.24e-03	p>0.05	p>0.05	p>0.05	p>0.05	0.47	57.78	1.12
**Parietal Lobe**												
BA1	3.8e-03	p<0.0001	-3.5e-05	p<0.0001	-1.46E-03	p>0.05	p>0.05	p>0.05	p>0.05	0.33	54.18	1.04
BA2	3.1e-03	p<0.0001	-2.9e-05	p<0.0001	1.92e-03	p>0.05	p>0.05	p>0.05	p>0.05	0.39	54.40	0.88
BA3	3.3e-03	p<0.0001	-3e-05	p<0.0001	5.69e-04	p>0.05	p>0.05	p>0.05	p>0.05	0.30	54.36	1.14
BA5	2.9e-03	p<0.0001	-2.7e-05	p<0.0001	2.23e-03	p>0.05	p>0.05	p>0.05	p>0.05	0.32	53.17	0.83
BA7	2.9e-03	p<0.0001	-2.7e-05	p<0.0001	1.64e-03	p>0.05	p>0.05	p>0.05	p>0.05	0.38	54.02	0.83
BA23	2.4e-03	p<0.0001	-2.1e-05	p<0.0001	-1.55E-03	p>0.05	p>0.05	p>0.05	p>0.05	0.38	57.20	1.28
BA29	2.5e-03	p<0.0001	-2.2e-05	p<0.0001	9.76e-05	p>0.05	p>0.05	p>0.05	p>0.05	0.26	56.38	1.49
BA30	2.3e-03	p<0.0001	-2e-05	p<0.0001	8.45e-05	p>0.05	p>0.05	p>0.05	p>0.05	0.30	57.78	1.65
BA31	2.4e-03	p<0.0001	-2.1e-05	p<0.0001	4.24e-04	p>0.05	p>0.05	p>0.05	p>0.05	0.37	56.44	1.14
BA39	2.6e-03	p<0.0001	-2.3e-05	p<0.0001	9.00e-04	p>0.05	p>0.05	p>0.05	p>0.05	0.41	56.61	1.10
BA40	2.7e-03	p<0.0001	-2.4e-05	p<0.0001	1.43e-03	p>0.05	p>0.05	p>0.05	p>0.05	0.45	56.32	1.00
**Temporal Lobe**												
BA20	1.8e-03	p<0.0001	-1.5e-05	p<0.0001	1.28e-03	p>0.05	p>0.05	p>0.05	p>0.05	0.37	59.63	1.81
BA21	2.2e-03	p<0.0001	-1.9e-05	p<0.0001	6.40e-04	p>0.05	p>0.05	p>0.05	p>0.05	0.40	57.78	1.27
BA22	2.6e-03	p<0.0001	-2.2e-05	p<0.0001	4.86e-04	p>0.05	p>0.05	p>0.05	p>0.05	0.48	58.91	1.22
BA37	2.1e-03	p<0.0001	-1.9e-05	p<0.0001	1.56e-04	p>0.05	p>0.05	p>0.05	p>0.05	0.35	57.37	1.39
BA38	1.8e-03	p<0.0001	-1.5e-05	p<0.0001	6.52e-04	p>0.05	p>0.05	p>0.05	p>0.05	0.38	60.53	1.71
BA41	2.4e-03	p<0.0001	-2.1e-05	p<0.0001	-1.36E-03	p>0.05	p>0.05	p>0.05	p>0.05	0.35	58.12	1.58
BA42	2.8e-03	p<0.0001	-2.5e-05	p<0.0001	4.22e-04	p>0.05	p>0.05	p>0.05	p>0.05	0.31	57.27	1.44
**Occipital Lobe**												
BA17	2.4e-03	p<0.0001	-2.3e-05	p<0.0001	-1.92E-03	p>0.05	p>0.05	p>0.05	p>0.05	0.20	53.19	1.27
BA18	2.5e-03	p<0.0001	-2.4e-05	p<0.0001	-3.54E-04	p>0.05	p>0.05	p>0.05	p>0.05	0.27	53.72	1.06
BA19	2.4e-03	p<0.0001	-2.2e-05	p<0.0001	-1.61E-04	p>0.05	p>0.05	p>0.05	p>0.05	0.32	54.75	1.03
**Mixed**												
BA26	2.5e-03	p<0.0001	-2.3e-05	p<0.0001	2.20e-04	p>0.05	p>0.05	p>0.05	p>0.05	0.13	55.99	2.25
BA27	2.1e-03	p<0.0001	-1.8e-05	p<0.0001	3.33e-03	p<0.05	p>0.05	p>0.05	p>0.05	0.30	59.75	2.23
BA28	3.5e-03	p<0.0001	-3.4e-05	p<0.0001	5.23e-03	p<0.01	p>0.05	p>0.05	p>0.05	0.30	52.65	0.83
BA35	2.1e-03	p<0.0001	-1.8e-05	p<0.0001	1.47e-03	p>0.05	p>0.05	p>0.05	p>0.05	0.30	58.44	1.80
BA36	2.6e-03	p<0.0001	-2.4e-05	p<0.0001	3.70e-03	p<0.01	p>0.05	p>0.05	p>0.05	0.36	52.92	0.84
BA43	2.8e-03	p<0.0001	-2.4e-05	p<0.0001	-1.46E-04	p>0.05	p>0.05	p<0.05	p>0.05	0.39	57.29	1.33
**R1-sWM**												
**Frontal Lobe**												
BA4	5.1e-03	p<0.0001	-6.2e-05	p<0.0001	4.12e-03	p>0.05	p>0.05	p>0.05	p>0.05	0.42	40.93	1.35
BA6	4.9e-03	p<0.0001	-6e-05	p<0.0001	4.53e-03	p>0.05	p>0.05	p>0.05	p>0.05	0.41	40.09	1.58
BA8	5.9e-03	p<0.0001	-7e-05	p<0.0001	5.16e-03	p>0.05	p>0.05	p>0.05	p>0.05	0.35	42.06	1.39
BA9	4.7e-03	p<0.0001	-5.9e-05	p<0.0001	5.72e-03	p>0.05	p<0.05	p>0.05	p>0.05	0.39	39.45	1.78
BA10	4.8e-03	p<0.0001	-6e-05	p<0.0001	5.97e-03	p>0.05	p>0.05	p>0.05	p>0.05	0.36	40.41	1.52
BA11	5.8e-03	p<0.0001	-6.8e-05	p<0.0001	8.94e-03	p<0.05	p<0.05	p>0.05	p>0.05	0.34	42.86	1.11
BA24	4e-03	p<0.0001	-5e-05	p<0.0001	1.32e-02	p<0.001	p>0.05	p>0.05	p>0.05	0.30	39.73	2.25
BA25	6.6e-03	p<0.0001	-7.6e-05	p<0.0001	8.04e-03	p>0.05	p>0.05	p>0.05	p>0.05	0.25	43.40	1.49
BA32	4.7e-03	p<0.0001	-5.8e-05	p<0.0001	4.99e-03	p>0.05	p>0.05	p>0.05	p>0.05	0.35	40.01	1.75
BA33	6e-03	p<0.0001	-6.7e-05	p<0.0001	1.16e-02	p<0.05	p>0.05	p>0.05	p>0.05	0.21	44.59	1.29
BA44	4e-03	p<0.0001	-5.2e-05	p<0.0001	1.02e-03	p>0.05	p>0.05	p>0.05	p>0.05	0.37	38.42	2.23
BA45	5e-03	p<0.0001	-5.9e-05	p<0.0001	1.61e-03	p>0.05	p>0.05	p>0.05	p>0.05	0.31	42.12	1.37
BA46	4.3e-03	p<0.0001	-5.4e-05	p<0.0001	1.01e-03	p>0.05	p>0.05	p>0.05	p>0.05	0.34	39.61	1.86

BA47	5.6e-03	p<0.0001	-6.5e-05	p<0.0001	4.19e-03	p>0.05	p>0.05	p>0.05	p>0.05	0.29	43.49	1.12
**Parietal Lobe**												
BA1	4.8e-03	p<0.0001	-5.2e-05	p<0.0001	3.34e-03	p>0.05	p>0.05	p>0.05	p>0.05	0.22	45.65	1.28
BA2	4.3e-03	p<0.0001	-5.2e-05	p<0.0001	4.29e-05	p>0.05	p>0.05	p>0.05	p>0.05	0.29	40.60	1.84
BA3	3.8e-03	p<0.0001	-4.2e-05	p<0.0001	5.30e-03	p>0.05	p>0.05	p>0.05	p>0.05	0.19	45.62	1.45
BA5	4.7e-03	p<0.0001	-5.7e-05	p<0.0001	5.24e-03	p>0.05	p>0.05	p>0.05	p>0.05	0.30	41.13	1.80
BA7	5e-03	p<0.0001	-5.8e-05	p<0.0001	3.86e-03	p>0.05	p>0.05	p>0.05	p>0.05	0.30	42.29	1.44
BA23	5.3e-03	p<0.0001	-6.4e-05	p<0.0001	9.80e-04	p>0.05	p>0.05	p>0.05	p>0.05	0.33	41.38	1.48
BA29	6.7e-03	p<0.0001	-7.3e-05	p<0.0001	1.08e-03	p>0.05	p>0.05	p>0.05	p>0.05	0.16	46.43	1.20
BA30	5.4e-03	p<0.0001	-6.4e-05	p<0.0001	3.44e-03	p>0.05	p>0.05	p>0.05	p>0.05	0.29	41.92	1.52
BA31	5.1e-03	p<0.0001	-6.2e-05	p<0.0001	2.57e-03	p>0.05	p>0.05	p>0.05	p>0.05	0.33	41.06	1.53
BA39	4.6e-03	p<0.0001	-5.6e-05	p<0.0001	8.73e-03	p<0.05	p>0.05	p>0.05	p>0.05	0.33	40.52	1.58
BA40	5.4e-03	p<0.0001	-6.3e-05	p<0.0001	9.85e-03	p<0.05	p>0.05	p>0.05	p>0.05	0.31	43.21	1.21
**Temporal Lobe**												
BA20	6.8e-03	p<0.0001	-7.4e-05	p<0.0001	5.09e-03	p>0.05	p>0.05	p>0.05	p>0.05	0.26	46.10	1.05
BA21	6.6e-03	p<0.0001	-7.3e-05	p<0.0001	1.17e-02	p<0.01	p>0.05	p>0.05	p>0.05	0.30	44.96	0.99
BA22	5.6e-03	p<0.0001	-6.3e-05	p<0.0001	3.14e-03	p>0.05	p>0.05	p>0.05	p>0.05	0.24	44.47	1.31
BA37	5.9e-03	p<0.0001	-6.8e-05	p<0.0001	7.53e-03	p>0.05	p>0.05	p>0.05	p>0.05	0.32	43.18	1.18
BA38	7.4e-03	p<0.0001	-7.5e-05	p<0.0001	8.22e-03	p>0.05	p>0.05	p>0.05	p>0.05	0.23	48.68	0.90
BA41	4e-03	p<0.0001	-5.2e-05	p<0.0001	-1.01E-04	p>0.05	p>0.05	p>0.05	p>0.05	0.35	38.61	1.98
BA42	3.3e-03	p<0.0001	-4.5e-05	p<0.0001	1.14e-03	p>0.05	p>0.05	p>0.05	p>0.05	0.36	35.99	2.93
**Occipital Lobe**												
BA17	2.4e-03	p<0.001	-2.9e-05	p<0.0001	5.11e-03	p>0.05	p>0.05	p<0.05	p>0.05	0.12	41.12	3.71
BA18	3.5e-03	p<0.0001	-4.1e-05	p<0.0001	4.09e-03	p>0.05	p>0.05	p>0.05	p>0.05	0.23	42.77	1.70
BA19	4.4e-03	p<0.0001	-5.4e-05	p<0.0001	4.49e-03	p>0.05	p>0.05	p>0.05	p>0.05	0.34	40.94	1.57
**Mixed**												
BA26	6.7e-03	p<0.0001	-7.1e-05	p<0.0001	2.21e-03	p>0.05	p>0.05	p>0.05	p>0.05	0.14	47.57	1.44
BA27	4.5e-03	p<0.0001	-3.9e-05	p<0.001	1.32e-02	p<0.05	p>0.05	p>0.05	p>0.05	0.09	56.93	3.65
BA28	5.5e-03	p<0.0001	-5.5e-05	p<0.0001	-1.85E-03	p>0.05	p>0.05	p>0.05	p>0.05	0.16	49.38	1.11
BA35	4.2e-03	p<0.0001	-4.6e-05	p<0.0001	6.44e-03	p>0.05	p>0.05	p>0.05	p>0.05	0.15	46.30	1.28
BA36	5.8e-03	p<0.0001	-5.8e-05	p<0.0001	-3.39E-03	p>0.05	p>0.05	p>0.05	p>0.05	0.20	50.46	0.97
BA43	5.6e-03	p<0.0001	-6.5e-05	p<0.0001	3.36e-03	p>0.05	p>0.05	p>0.05	p>0.05	0.29	42.52	1.31
**R1-WM bundles**												
ARCL	4.3e-03	p<0.0001	-5.3e-05	p<0.0001	8.17e-03	p<0.05	p>0.05	p>0.05	p>0.05	0.29	40.23	2.16
ARCR	4.2e-03	p<0.0001	-5.3e-05	p<0.0001	6.71e-03	p>0.05	p>0.05	p>0.05	p>0.05	0.30	39.62	2.18
ATRL	5.1e-03	p<0.0001	-6.5e-05	p<0.0001	5.43e-03	p>0.05	p>0.05	p>0.05	p>0.05	0.38	38.76	1.97
ATRR	5.1e-03	p<0.0001	-6.5e-05	p<0.0001	6.79e-03	p>0.05	p>0.05	p>0.05	p>0.05	0.35	39.47	1.89
CGCL	4.8e-03	p<0.0001	-6e-05	p<0.0001	7.15e-03	p>0.05	p>0.05	p>0.05	p>0.05	0.31	40.32	1.83
CGCR	4.8e-03	p<0.0001	-6e-05	p<0.0001	1.08e-02	p<0.01	p>0.05	p>0.05	p>0.05	0.34	39.97	1.90
CSTL	4.1e-03	p<0.0001	-5.2e-05	p<0.0001	5.76e-03	p>0.05	p>0.05	p>0.05	p>0.05	0.37	39.08	2.20
CSTR	4.2e-03	p<0.0001	-5.3e-05	p<0.0001	6.13e-03	p>0.05	p>0.05	p>0.05	p>0.05	0.38	39.12	2.06
FA	4.1e-03	p<0.0001	-5.6e-05	p<0.0001	5.97e-03	p>0.05	p>0.05	p>0.05	p>0.05	0.36	36.14	2.83
FP	3.2e-03	p<0.001	-4.8e-05	p<0.0001	-4.36E-03	p>0.05	p>0.05	p>0.05	p>0.05	0.40	32.77	3.79
IFOL	4e-03	p<0.0001	-5.6e-05	p<0.0001	2.80e-03	p>0.05	p>0.05	p>0.05	p>0.05	0.43	34.77	2.96
IFOR	3.9e-03	p<0.0001	-5.5e-05	p<0.0001	2.64e-03	p>0.05	p>0.05	p>0.05	p>0.05	0.40	35.32	2.93
ILFL	4e-03	p<0.0001	-5.5e-05	p<0.0001	4.64e-03	p>0.05	p>0.05	p>0.05	p>0.05	0.38	36.44	2.55
ILFR	4.3e-03	p<0.0001	-5.6e-05	p<0.0001	2.26e-03	p>0.05	p>0.05	p>0.05	p>0.05	0.37	37.67	2.28
pARCL	4.6e-03	p<0.0001	-5.5e-05	p<0.0001	9.00e-03	p<0.05	p>0.05	p>0.05	p>0.05	0.24	41.60	1.88
pARCR	4.6e-03	p<0.0001	-5.6e-05	p<0.0001	7.33e-03	p>0.05	p>0.05	p>0.05	p>0.05	0.28	40.63	2.04
SLFL	4.8e-03	p<0.0001	-5.8e-05	p<0.0001	8.52e-03	p<0.05	p>0.05	p>0.05	p>0.05	0.32	40.96	1.91
SLFR	4.9e-03	p<0.0001	-6.1e-05	p<0.0001	8.65e-03	p<0.05	p>0.05	p>0.05	p>0.05	0.35	40.23	1.92
VOFR	5.3e-03	p<0.0001	-6.4e-05	p<0.0001	6.22e-03	p>0.05	p>0.05	p>0.05	p>0.05	0.35	41.71	1.50
VOFL	4.1e-03	p<0.0001	-5.1e-05	p<0.0001	1.22e-02	p<0.01	p>0.05	p>0.05	p>0.05	0.30	39.78	2.10
UNCR	6.3e-03	p<0.0001	-7e-05	p<0.0001	9.68e-03	p>0.05	p>0.05	p>0.05	p>0.05	0.13	44.74	1.81
UNCL	4.9e-03	p<0.001	-5.6e-05	p<0.0001	1.11e-02	p>0.05	p>0.05	p>0.05	p>0.05	0.10	43.17	3.45
**R2*-cGM**												
**Frontal Lobe**												
BA4	1.7e-01	p<0.0001	-1.4e-03	p<0.0001	5.26e-02	p>0.05	p>0.05	p>0.05	p>0.05	0.35	61.36	2.24
BA6	1.4e-01	p<0.0001	-1.2e-03	p<0.0001	1.86e-01	p<0.05	p>0.05	p>0.05	p>0.05	0.39	61.91	2.17
BA8	1.4e-01	p<0.0001	-1.1e-03	p<0.0001	1.83e-01	p<0.05	p>0.05	p>0.05	p>0.05	0.35	60.37	2.01
BA9	1.3e-01	p<0.0001	-1.2e-03	p<0.0001	1.24e-01	p>0.05	p>0.05	p>0.05	p>0.05	0.29	57.21	1.55
BA10	1.2e-01	p<0.0001	-1.1e-03	p<0.0001	2.25e-01	p<0.05	p>0.05	p>0.05	p>0.05	0.19	56.67	1.86
BA11	1.2e-01	p<0.0001	-9.6e-04	p<0.0001	3.37e-01	p<0.01	p>0.05	p>0.05	p>0.05	0.17	61.27	4.00
BA24	1.2e-01	p<0.0001	-9.5e-04	p<0.0001	2.44e-01	p<0.05	p>0.05	p>0.05	p>0.05	0.18	61.51	3.46
BA25	9.9e-02	p<0.05	-8.6e-04	p<0.05	8.63e-02	p>0.05	p>0.05	p>0.05	p>0.05	0.04	58.14	4.09

BA32	1.4e-01	p<0.0001	-1.2e-03	p<0.0001	2.29e-01	p<0.05	p>0.05	p>0.05	p>0.05	0.24	57.29	1.79
BA33	6.3e-02	p<0.05	-5e-04	p<0.05	3.96e-01	p<0.01	p>0.05	p>0.05	p>0.05	0.08	64.64	7.19
BA44	1.3e-01	p<0.0001	-1.1e-03	p<0.0001	1.71e-01	p>0.05	p>0.05	p>0.05	p>0.05	0.32	62.28	2.87
BA45	1.3e-01	p<0.0001	-1.1e-03	p<0.0001	1.14e-01	p>0.05	p>0.05	p>0.05	p>0.05	0.21	59.58	2.58
BA46	1.3e-01	p<0.0001	-1.1e-03	p<0.0001	1.00e-01	p>0.05	p>0.05	p>0.05	p>0.05	0.24	59.86	2.41
BA47	1.1e-01	p<0.0001	-8.5e-04	p<0.001	2.13e-01	p>0.05	p>0.05	p>0.05	p>0.05	0.14	64.11	3.72
**Parietal Lobe**												
BA1	1.3e-01	p<0.0001	-1.2e-03	p<0.0001	-1.10E-01	p>0.05	p>0.05	p>0.05	p>0.05	0.17	55.67	1.73
BA2	1.4e-01	p<0.0001	-1.2e-03	p<0.0001	7.64e-02	p>0.05	p>0.05	p>0.05	p>0.05	0.21	56.90	1.75
BA3	8.6e-02	p<0.0001	-7.7e-04	p<0.0001	1.13e-01	p>0.05	p>0.05	p>0.05	p>0.05	0.08	55.77	4.17
BA5	1.1e-01	p<0.0001	-9.7e-04	p<0.0001	1.94e-01	p<0.05	p>0.05	p>0.05	p>0.05	0.20	57.71	2.00
BA7	1.2e-01	p<0.0001	-1e-03	p<0.0001	1.17e-01	p>0.05	p>0.05	p>0.05	p>0.05	0.22	58.08	2.17
BA23	1.3e-01	p<0.0001	-1.1e-03	p<0.0001	-3.98E-02	p>0.05	p>0.05	p>0.05	p>0.05	0.13	59.76	3.55
BA29	6.5e-02	p>0.05	-4.1e-04	p>0.05	2.76e-01	p>0.05	p>0.05	p>0.05	p>0.05	-	-	-
BA30	1.1e-01	p<0.0001	-9.3e-04	p<0.001	1.21e-01	p>0.05	p>0.05	p>0.05	p>0.05	0.11	59.29	3.58
BA31	1.4e-01	p<0.0001	-1.2e-03	p<0.0001	1.05e-01	p>0.05	p>0.05	p>0.05	p>0.05	0.16	58.73	2.33
BA39	1.4e-01	p<0.0001	-1.2e-03	p<0.0001	7.46e-02	p>0.05	p>0.05	p>0.05	p>0.05	0.23	59.26	2.19
BA40	1.3e-01	p<0.0001	-1.1e-03	p<0.0001	1.03e-01	p>0.05	p>0.05	p>0.05	p>0.05	0.25	60.17	2.49
**Temporal Lobe**												
BA20	1.1e-01	p<0.0001	-7.7e-04	p<0.01	2.12e-01	p>0.05	p>0.05	p>0.05	p>0.05	0.20	70.64	5.86
BA21	1.2e-01	p<0.0001	-8.8e-04	p<0.001	3.01e-01	p<0.05	p>0.05	p>0.05	p>0.05	0.20	65.94	3.96
BA22	1.4e-01	p<0.0001	-1.1e-03	p<0.0001	1.66e-01	p>0.05	p>0.05	p<0.05	p>0.05	0.33	61.69	2.42
BA37	1.5e-01	p<0.0001	-1.2e-03	p<0.0001	1.58e-01	p>0.05	p>0.05	p>0.05	p>0.05	0.25	60.20	2.10
BA38	8.4e-02	p<0.001	-6.2e-04	p<0.01	3.52e-01	p<0.01	p>0.05	p>0.05	p>0.05	0.17	69.42	7.06
BA41	1.4e-01	p<0.0001	-1.1e-03	p<0.0001	-4.64E-02	p>0.05	p>0.05	p>0.05	p>0.05	0.19	59.35	2.68
BA42	1.6e-01	p<0.0001	-1.4e-03	p<0.0001	2.33e-01	p<0.05	p>0.05	p>0.05	p>0.05	0.26	59.77	2.26
**Occipital Lobe**												
BA17	1.1e-01	p<0.0001	-1e-03	p<0.0001	8.83e-02	p>0.05	p>0.05	p>0.05	p>0.05	0.09	56.13	2.54
BA18	1.4e-01	p<0.0001	-1.2e-03	p<0.0001	1.55e-01	p>0.05	p>0.05	p>0.05	p>0.05	0.20	58.26	2.13
BA19	1.3e-01	p<0.0001	-1e-03	p<0.0001	8.68e-02	p>0.05	p>0.05	p>0.05	p>0.05	0.22	59.86	2.60
**Mixed**												
BA26	4.9e-02	p>0.05	-1.3e-04	p>0.05	2.31e-01	p>0.05	p>0.05	p>0.05	p<0.05	-	-	-
BA27	1.3e-01	p<0.0001	-1.1e-03	p<0.001	-1.17E-02	p>0.05	p>0.05	p>0.05	p>0.05	0.08	58.04	5.06
BA28	1.1e-01	p<0.001	-9.3e-04	p<0.01	4.31e-01	p<0.01	p>0.05	p>0.05	p>0.05	0.08	59.03	8.50
BA35	1.1e-01	p<0.01	-8.3e-04	p<0.05	-8.27E-02	p>0.05	p>0.05	p>0.05	p>0.05	0.10	66.41	6.23
BA36	1.3e-01	p<0.0001	-1.1e-03	p<0.001	1.72e-01	p>0.05	p>0.05	p>0.05	p>0.05	0.10	59.80	4.33
BA43	1.4e-01	p<0.0001	-1.2e-03	p<0.0001	1.16e-01	p>0.05	p>0.05	p>0.05	p>0.05	0.28	59.72	2.22
**R2*-sWM**												
**Frontal Lobe**												
BA4	1.7e-01	p<0.0001	-1.6e-03	p<0.0001	2.89e-01	p<0.01	p>0.05	p>0.05	p<0.05	0.29	54.14	1.12
BA6	1.6e-01	p<0.0001	-1.5e-03	p<0.0001	2.31e-01	p<0.01	p>0.05	p>0.05	p>0.05	0.27	52.69	1.04
BA8	1.7e-01	p<0.0001	-1.7e-03	p<0.0001	2.17e-01	p<0.05	p>0.05	p>0.05	p<0.05	0.24	50.84	0.94
BA9	1.6e-01	p<0.0001	-1.6e-03	p<0.0001	1.87e-01	p<0.05	p>0.05	p>0.05	p>0.05	0.23	49.05	0.86
BA10	1.5e-01	p<0.0001	-1.5e-03	p<0.0001	2.66e-01	p<0.01	p>0.05	p>0.05	p>0.05	0.21	47.70	1.00
BA11	1.4e-01	p<0.0001	-1.4e-03	p<0.0001	3.31e-01	p<0.01	p>0.05	p>0.05	p>0.05	0.15	49.86	1.35
BA24	1.3e-01	p<0.0001	-1.3e-03	p<0.0001	5.33e-01	p<0.0001	p>0.05	p>0.05	p<0.05	0.20	51.40	1.32
BA25	1.1e-01	p<0.01	-1.2e-03	p<0.001	3.51e-01	p<0.05	p>0.05	p>0.05	p<0.05	0.09	44.44	4.76
BA32	1.7e-01	p<0.0001	-1.8e-03	p<0.0001	3.45e-01	p<0.001	p>0.05	p>0.05	p<0.05	0.26	48.35	0.84
BA33	1.3e-01	p<0.0001	-1.4e-03	p<0.0001	4.88e-01	p<0.001	p>0.05	p>0.05	p<0.05	0.15	47.45	1.53
BA44	1.3e-01	p<0.0001	-1.3e-03	p<0.0001	2.62e-01	p<0.05	p>0.05	p>0.05	p>0.05	0.16	51.75	1.41
BA45	1.4e-01	p<0.0001	-1.3e-03	p<0.0001	2.10e-01	p>0.05	p>0.05	p>0.05	p>0.05	0.10	50.79	1.47
BA46	1.3e-01	p<0.0001	-1.3e-03	p<0.0001	1.69e-01	p>0.05	p>0.05	p>0.05	p>0.05	0.14	49.33	1.19
BA47	1.5e-01	p<0.0001	-1.5e-03	p<0.0001	2.84e-01	p<0.05	p>0.05	p>0.05	p>0.05	0.09	50.16	1.57
**Parietal Lobe**												
BA1	1.5e-01	p<0.0001	-1.4e-03	p<0.0001	1.03e-01	p>0.05	p>0.05	p>0.05	p>0.05	0.23	55.22	1.33
BA2	1.4e-01	p<0.0001	-1.3e-03	p<0.0001	2.34e-01	p<0.05	p>0.05	p>0.05	p<0.05	0.20	53.50	1.34
BA3	1.3e-01	p<0.0001	-1.2e-03	p<0.0001	2.13e-01	p<0.05	p>0.05	p>0.05	p>0.05	0.23	56.12	1.69
BA5	1.3e-01	p<0.0001	-1.3e-03	p<0.0001	3.63e-01	p<0.001	p>0.05	p>0.05	p<0.05	0.19	51.96	1.29
BA7	1.4e-01	p<0.0001	-1.3e-03	p<0.0001	3.31e-01	p<0.001	p>0.05	p>0.05	p<0.05	0.21	52.60	1.26
BA23	1.5e-01	p<0.0001	-1.5e-03	p<0.0001	7.19e-02	p>0.05	p<0.05	p>0.05	p>0.05	0.10	49.23	1.31
BA29	1e-01	p<0.01	-9.6e-04	p<0.01	3.01e-01	p>0.05	p>0.05	p>0.05	p>0.05	0.05	53.90	7.74
BA30	1.2e-01	p<0.0001	-1.2e-03	p<0.0001	2.68e-01	p<0.05	p>0.05	p>0.05	p>0.05	0.09	49.33	1.68
BA31	1.5e-01	p<0.0001	-1.4e-03	p<0.0001	3.63e-01	p<0.01	p>0.05	p>0.05	p>0.05	0.15	51.62	1.25
BA39	1.5e-01	p<0.0001	-1.4e-03	p<0.0001	3.55e-01	p<0.001	p>0.05	p>0.05	p>0.05	0.20	51.87	1.17
BA40	1.4e-01	p<0.0001	-1.4e-03	p<0.0001	3.60e-01	p<0.001	p>0.05	p>0.05	p>0.05	0.20	52.05	1.23

**Temporal Lobe**												
BA20	1.4e-01	p<0.0001	-1.3e-03	p<0.0001	1.95e-01	p>0.05	p>0.05	p>0.05	p>0.05	0.14	54.41	1.93
BA21	1.7e-01	p<0.0001	-1.6e-03	p<0.0001	3.20e-01	p<0.05	p>0.05	p>0.05	p>0.05	0.16	51.95	1.29
BA22	1.7e-01	p<0.0001	-1.6e-03	p<0.0001	2.35e-01	p<0.05	p>0.05	p>0.05	p>0.05	0.22	52.22	1.02
BA37	1.7e-01	p<0.0001	-1.6e-03	p<0.0001	2.09e-01	p>0.05	p>0.05	p>0.05	p<0.05	0.20	51.40	1.03
BA38	1.4e-01	p<0.0001	-1.3e-03	p<0.0001	3.12e-01	p<0.05	p>0.05	p>0.05	p>0.05	0.13	53.54	1.93
BA41	1.3e-01	p<0.0001	-1.3e-03	p<0.0001	6.45e-02	p>0.05	p>0.05	p>0.05	p>0.05	0.11	50.30	1.32
BA42	1.5e-01	p<0.0001	-1.4e-03	p<0.0001	2.90e-01	p<0.01	p>0.05	p>0.05	p<0.05	0.21	54.23	1.37
**Occipital Lobe**												
BA17	1.2e-01	p<0.0001	-1e-03	p<0.0001	2.24e-01	p<0.05	p<0.05	p>0.05	p>0.05	0.13	56.31	2.17
BA18	1.5e-01	p<0.0001	-1.3e-03	p<0.0001	3.23e-01	p<0.01	p>0.05	p>0.05	p>0.05	0.23	56.31	1.60
BA19	1.4e-01	p<0.0001	-1.3e-03	p<0.0001	3.09e-01	p<0.01	p>0.05	p>0.05	p<0.05	0.20	53.49	1.40
**Mixed**												
BA26	8.7e-02	p<0.05	-7.8e-04	p<0.05	3.59e-01	p<0.05	p>0.05	p>0.05	p>0.05	0.04	55.95	4.54
BA27	1.3e-01	p<0.0001	-1.1e-03	p<0.001	2.33e-01	p>0.05	p>0.05	p>0.05	p>0.05	0.10	58.68	4.88
BA28	1.6e-01	p<0.0001	-1.4e-03	p<0.0001	3.14e-01	p<0.05	p>0.05	p>0.05	p>0.05	0.13	55.20	2.42
BA35	1.3e-01	p<0.0001	-1.2e-03	p<0.001	1.58e-01	p>0.05	p>0.05	p>0.05	p>0.05	0.07	55.72	3.24
BA36	1.3e-01	p<0.0001	-1.2e-03	p<0.0001	1.13e-01	p>0.05	p>0.05	p>0.05	p>0.05	0.11	57.00	3.07
BA43	1.6e-01	p<0.0001	-1.5e-03	p<0.0001	1.82e-01	p>0.05	p>0.05	p>0.05	p>0.05	0.19	51.25	1.07
**R2*-WM bundles**												
ARCL	1.3e-01	p<0.0001	-1.4e-03	p<0.0001	2.43e-01	p<0.05	p>0.05	p>0.05	p>0.05	0.13	46.56	1.35
ARCR	7e-02	p<0.01	-7.8e-04	p<0.001	4.06e-01	p<0.001	p>0.05	p>0.05	p<0.01	0.10	44.01	4.39
ATRL	1.3e-01	p<0.0001	-1.6e-03	p<0.0001	2.21e-01	p>0.05	p>0.05	p>0.05	p>0.05	0.23	41.86	1.77
ATRR	1.3e-01	p<0.0001	-1.5e-03	p<0.0001	3.89e-01	p<0.01	p>0.05	p>0.05	p>0.05	0.20	42.56	1.76
CGCL	1.3e-01	p<0.0001	-1.4e-03	p<0.0001	3.90e-01	p<0.01	p>0.05	p>0.05	p<0.05	0.15	47.28	1.46
CGCR	1.2e-01	p<0.0001	-1.3e-03	p<0.0001	3.49e-01	p<0.01	p>0.05	p>0.05	p<0.05	0.12	47.18	1.57
CSTL	1.3e-01	p<0.0001	-1.3e-03	p<0.0001	2.77e-01	p<0.05	p>0.05	p>0.05	p>0.05	0.12	47.47	1.34
CSTR	8e-02	p<0.001	-8.2e-04	p<0.001	5.27e-01	p<0.0001	p>0.05	p>0.05	p>0.05	0.11	48.77	2.57
FA	7.3e-02	p<0.01	-9.6e-04	p<0.001	3.50e-01	p<0.05	p>0.05	p>0.05	p<0.01	0.15	37.28	5.00
FP	4.4e-02	p>0.05	-7e-04	p<0.05	2.18e-02	p>0.05	p>0.05	p>0.05	p>0.05	0.10	28.51	9.16
IFOL	1.1e-01	p<0.0001	-1.3e-03	p<0.0001	2.68e-01	p<0.05	p<0.05	p>0.05	p<0.05	0.18	42.49	1.87
IFOR	9.1e-02	p<0.001	-1.1e-03	p<0.0001	3.17e-01	p<0.05	p>0.05	p>0.05	p<0.001	0.20	39.67	2.74
ILFL	1.1e-01	p<0.0001	-1.3e-03	p<0.0001	1.80e-01	p>0.05	p>0.05	p>0.05	p>0.05	0.10	45.14	1.75
ILFR	8.7e-02	p<0.001	-1.1e-03	p<0.0001	3.24e-01	p<0.05	p>0.05	p>0.05	p<0.01	0.15	40.81	3.01
pARCL	1.4e-01	p<0.0001	-1.4e-03	p<0.0001	3.10e-01	p<0.01	p>0.05	p>0.05	p>0.05	0.13	49.99	1.28
pARCR	1.2e-01	p<0.0001	-1.2e-03	p<0.0001	4.03e-01	p<0.01	p>0.05	p>0.05	p<0.05	0.12	48.35	1.64
SLFL	1.3e-01	p<0.0001	-1.4e-03	p<0.0001	2.53e-01	p<0.05	p>0.05	p>0.05	p<0.05	0.14	47.53	1.28
SLFR	8.9e-02	p<0.0001	-9.6e-04	p<0.0001	4.53e-01	p<0.0001	p>0.05	p>0.05	p<0.001	0.16	46.10	2.06
VOFR	1.3e-01	p<0.0001	-1.3e-03	p<0.0001	4.45e-01	p<0.001	p>0.05	p>0.05	p>0.05	0.14	50.92	1.39
VOFL	1.3e-01	p<0.0001	-1.3e-03	p<0.0001	2.65e-01	p<0.05	p<0.05	p>0.05	p<0.05	0.14	51.35	1.42
UNCR	1.3e-01	p<0.0001	-1.4e-03	p<0.0001	2.47e-01	p>0.05	p>0.05	p>0.05	p>0.05	0.10	49.13	1.66
UNCL	1.3e-01	p<0.0001	-1.3e-03	p<0.0001	4.18e-01	p<0.01	p>0.05	p>0.05	p>0.05	0.10	49.77	1.95
**Susceptibility-cGM**												
**Frontal Lobe**												
BA4	3.2e-01	p<0.0001	-2.8e-03	p<0.0001	-4.66E-04	p>0.05	p>0.05	p>0.05	p<0.05	0.19	56.52	2.14
BA6	2.2e-01	p<0.0001	-1.9e-03	p<0.001	7.17e-05	p>0.05	p>0.05	p>0.05	p<0.05	0.08	57.46	3.94
BA8	1.4e-01	p<0.01	-1.1e-03	p<0.05	3.21e-04	p>0.05	p>0.05	p>0.05	p>0.05	0.06	63.88	6.14
BA9	1.1e-01	p<0.01	-1e-03	p<0.05	2.65e-04	p>0.05	p<0.05	p>0.05	p>0.05	0.04	57.69	3.81
BA10	4.8e-02	p>0.05	-4.8e-04	p>0.05	-2.11E-04	p>0.05	p>0.05	p>0.05	p>0.05	-	-	-
BA11	2.8e-02	p>0.05	-3.1e-04	p>0.05	-1.64E-04	p>0.05	p>0.05	p>0.05	p>0.05	-	-	-
BA24	1.4e-01	p<0.01	-1.5e-03	p<0.01	3.09e-04	p>0.05	p>0.05	p>0.05	p<0.05	0.06	45.36	3.33
BA25	6.1e-04	p>0.05	-1.1e-04	p>0.05	5.77e-04	p>0.05	p>0.05	p>0.05	p>0.05	-	-	-
BA32	2e-01	p<0.0001	-1.9e-03	p<0.001	3.30e-04	p>0.05	p>0.05	p>0.05	p>0.05	0.05	53.49	3.82
BA33	-6.1e-03	p>0.05	-2.4e-04	p>0.05	3.81e-04	p>0.05	p>0.05	p>0.05	p>0.05	-	-	-
BA44	1.7e-01	p<0.01	-1.5e-03	p<0.05	2.65e-04	p>0.05	p>0.05	p>0.05	p>0.05	0.05	57.74	3.95
BA45	1.1e-01	p<0.05	-9.4e-04	p<0.05	-1.63E-04	p>0.05	p>0.05	p<0.05	p<0.0001	0.08	59.07	6.19
BA46	8.8e-02	p<0.05	-8.5e-04	p<0.05	-2.03E-04	p>0.05	p>0.05	p>0.05	p<0.05	0.03	51.92	3.70
BA47	7.5e-02	p>0.05	-5.6e-04	p>0.05	-1.23E-04	p>0.05	p>0.05	p>0.05	p>0.05	-	-	-
**Parietal Lobe**												
BA1		p<0.0001	-1.3e-03	p<0.0001	-1.87E-04	p>0.05	p>0.05	p>0.05	p>0.05	0.09	57.87	5.19
BA2	2.6e-01	p<0.0001	-2.6e-03	p<0.0001	-2.31E-04	p>0.05	p>0.05	p>0.05	p>0.05	0.10	51.09	1.48
BA3	4e-02	p>0.05	-6.4e-04	p>0.05	-2.99E-04	p>0.05	p>0.05	p>0.05	p>0.05	-	-	-
BA5	2.7e-01	p<0.0001	-2.4e-03	p<0.0001	7.09e-05	p>0.05	p>0.05	p>0.05	p>0.05	0.11	56.69	2.62
BA7	1.9e-01	p<0.0001	-1.9e-03	p<0.0001	-1.44E-04	p>0.05	p>0.05	p>0.05	p<0.05	0.09	51.21	1.80
BA23	3.4e-01	p<0.0001	-3e-03	p<0.0001	-2.77E-04	p>0.05	p>0.05	p>0.05	p<0.05	0.15	57.49	2.90

BA29	1.9e-01	p>0.05	-2.1e-03	p>0.05	-3.65E-04	p>0.05	p>0.05	p>0.05	p>0.05	-	-	-
BA30	1.3e-01	p<0.05	-1.4e-03	p<0.05	-9.04E-04	p<0.01	p>0.05	p>0.05	p>0.05	0.05	47.23	3.37
BA31	2.7e-01	p<0.0001	-2.5e-03	p<0.0001	2.96e-05	p>0.05	p>0.05	p>0.05	p<0.001	0.12	53.69	2.11
BA39	1.6e-01	p<0.001	-1.6e-03	p<0.001	-2.91E-04	p>0.05	p>0.05	p>0.05	p>0.05	0.07	49.61	3.53
BA40	1.8e-01	p<0.0001	-1.8e-03	p<0.0001	-1.02E-04	p>0.05	p>0.05	p>0.05	p>0.05	0.09	52.19	1.83
**Temporal Lobe**												
BA20		p>0.05	-2.4e-04	p>0.05	-2.07E-04	p>0.05	p>0.05	p>0.05	p>0.05	-	-	-
BA21	3.4e-02	p>0.05	-3.2e-04	p>0.05	-1.59E-04	p>0.05	p>0.05	p>0.05	p>0.05	-	-	-
BA22	1.6e-01	p<0.001	-1.6e-03	p<0.0001	-3.45E-04	p>0.05	p>0.05	p>0.05	p>0.05	0.06	48.49	2.21
BA37	7.6e-02	p<0.05	-8.7e-04	p<0.05	-4.47E-04	p<0.05	p>0.05	p>0.05	p>0.05	0.06	43.43	2.99
BA38	2.7e-02	p>0.05	-3.3e-04	p>0.05	-7.23E-04	p<0.001	p>0.05	p>0.05	p>0.05	-	-	-
BA41	2.2e-01	p<0.001	-2.6e-03	p<0.0001	-5.93E-04	p>0.05	p>0.05	p>0.05	p>0.05	0.13	41.42	2.88
BA42	2.3e-01	p<0.0001	-2.3e-03	p<0.0001	-9.06E-04	p<0.01	p>0.05	p>0.05	p>0.05	0.09	51.52	2.28
**Occipital Lobe**												
BA17		p<0.05	-8.3e-04	p<0.05	-7.85E-05	p>0.05	p>0.05	p>0.05	p<0.01	0.03	47.75	3.95
BA18	1.1e-01	p<0.001	-1.2e-03	p<0.0001	-4.24E-04	p<0.01	p>0.05	p>0.05	p<0.01	0.11	46.24	3.84
BA19	1.4e-01	p<0.001	-1.5e-03	p<0.0001	-5.11E-04	p<0.01	p>0.05	p>0.05	p<0.05	0.10	45.60	3.16
**Mixed**												
BA26	1.2e-01	p>0.05	-1.1e-03	p>0.05	3.64e-04	p>0.05	p>0.05	p>0.05	p>0.05	-	-	-
BA27	-2.8e-03	p>0.05	2.1e-04	p>0.05	-6.08E-04	p>0.05	p>0.05	p>0.05	p<0.05	-	-	-
BA28	4.9e-02	p>0.05	-3.7e-04	p>0.05	2.49e-04	p>0.05	p>0.05	p>0.05	p<0.05	-	-	-
BA35	-2.9e-02	p>0.05	2.7e-04	p>0.05	-1.21E-03	p<0.001	p>0.05	p>0.05	p>0.05	-	-	-
BA36	4.2e-02	p>0.05	-2.9e-04	p>0.05	-2.51E-04	p>0.05	p>0.05	p>0.05	p>0.05	-	-	-
BA43	1.5e-01	p<0.01	-1.9e-03	p<0.001	1.22e-04	p>0.05	p>0.05	p>0.05	p>0.05	0.10	40.36	3.15
**Susceptibility-sWM**												
**Frontal Lobe**												
BA4	1.1e-01	p<0.05	-6.4e-04	p>0.05	-6.11E-06	p>0.05	p>0.05	p>0.05	p>0.05	-	-	-
BA6	1.8e-01	p<0.0001	-1.7e-03	p<0.0001	-4.13E-04	p>0.05	p<0.05	p>0.05	p>0.05	0.09	53.51	2.91
BA8	1.5e-01	p<0.001	-1.5e-03	p<0.001	-7.36E-04	p<0.001	p<0.05	p>0.05	p>0.05	0.08	51.39	6.08
BA9	1.6e-01	p<0.001	-1.7e-03	p<0.0001	-6.81E-04	p<0.01	p>0.05	p>0.05	p>0.05	0.09	45.52	2.32
BA10	1.4e-01	p<0.01	-1.8e-03	p<0.001	-7.40E-04	p<0.01	p>0.05	p>0.05	p>0.05	0.14	38.12	3.08
BA11	1e-02	p>0.05	-2.9e-04	p>0.05	-3.49E-04	p<0.05	p>0.05	p>0.05	p>0.05	-	-	-
BA24	3.7e-01	p<0.0001	-3.8e-03	p<0.0001	-1.29E-04	p>0.05	p>0.05	p>0.05	p>0.05	0.10	49.29	1.42
BA25	1e-01	p>0.05	-1.5e-03	p<0.05	1.80e-04	p>0.05	p>0.05	p>0.05	p>0.05	0.09	33.60	6.22
BA32	3.2e-01	p<0.0001	-3.3e-03	p<0.0001	7.14e-05	p>0.05	p>0.05	p>0.05	p>0.05	0.10	48.03	1.47
BA33	2.1e-01	p<0.01	-2.5e-03	p<0.001	-2.82E-04	p>0.05	p>0.05	p>0.05	p>0.05	0.07	40.98	4.15
BA44	1e-01	p<0.05	-1.1e-03	p<0.05	-3.61E-04	p>0.05	p<0.05	p>0.05	p>0.05	0.03	45.61	4.21
BA45	8.1e-02	p>0.05	-9.5e-04	p>0.05	-2.19E-04	p>0.05	p>0.05	p>0.05	p>0.05	-	-	-
BA46	1.1e-01	p<0.05	-1.4e-03	p<0.01	-7.44E-04	p<0.01	p>0.05	p>0.05	p>0.05	0.08	39.44	4.40
BA47	-3.7e-02	p>0.05	7.2e-05	p>0.05	-1.79E-04	p>0.05	p>0.05	p>0.05	p>0.05	-	-	-
**Parietal Lobe**												
BA1	5.8e-02	p>0.05	-1.9e-04	p>0.05	1.21e-04	p>0.05	p>0.05	p>0.05	p>0.05	-	-	-
BA2	1.5e-01	p<0.01	-1.2e-03	p<0.01	-1.53E-04	p>0.05	p>0.05	p>0.05	p>0.05	0.06	59.93	3.78
BA3	6e-02	p>0.05	-4.2e-04	p>0.05	-4.21E-04	p<0.05	p>0.05	p>0.05	p>0.05	-	-	-
BA5	2.1e-01	p<0.001	-1.7e-03	p<0.01	-9.28E-05	p>0.05	p>0.05	p>0.05	p>0.05	0.07	60.40	3.79
BA7	7.5e-02	p>0.05	-6.8e-04	p>0.05	4.29e-05	p>0.05	p>0.05	p>0.05	p>0.05	-	-	-
BA23	4.1e-01	p<0.0001	-3.9e-03	p<0.0001	-8.18E-04	p<0.05	p>0.05	p>0.05	p>0.05	0.09	52.74	1.93
BA29	2.1e-01	p>0.05	-2.1e-03	p>0.05	-9.16E-04	p>0.05	p>0.05	p>0.05	p>0.05	-	-	-
BA30	-5e-02	p>0.05	4.3e-04	p>0.05	-2.88E-04	p>0.05	p>0.05	p>0.05	p>0.05	-	-	-
BA31	2.6e-01	p<0.001	-2.5e-03	p<0.001	3.32e-04	p>0.05	p>0.05	p>0.05	p>0.05	0.04	52.00	3.16
BA39	-6.1e-03	p>0.05	7.4e-05	p>0.05	-1.26E-04	p>0.05	p>0.05	p>0.05	p>0.05	-	-	-
BA40	4.3e-02	p>0.05	-4.3e-04	p>0.05	-2.03E-04	p>0.05	p>0.05	p>0.05	p>0.05	-	-	-
**Temporal Lobe**												
BA20	2.5e-02	p>0.05	-1.6e-04	p>0.05	-1.30E-04	p>0.05	p>0.05	p>0.05	p>0.05	-	-	-
BA21	-1.6e-02	p>0.05	1.1e-04	p>0.05	-5.54E-04	p<0.01	p>0.05	p>0.05	p>0.05	-	-	-
BA22	3.1e-02	p>0.05	-5.5e-04	p>0.05	-5.93E-04	p<0.05	p>0.05	p>0.05	p>0.05	-	-	-
BA37	-1.8e-02	p>0.05	1.4e-04	p>0.05	-6.06E-04	p<0.01	p>0.05	p>0.05	p>0.05	-	-	-
BA38	2.8e-02	p>0.05	-4.1e-04	p>0.05	-7.82E-04	p<0.001	p>0.05	p>0.05	p>0.05	-	-	-
BA41	-2.2e-02	p>0.05	3.5e-04	p>0.05	1.68e-05	p>0.05	p>0.05	p>0.05	p>0.05	-	-	-
BA42	1.9e-01	p<0.001	-1.6e-03	p<0.01	-8.33E-04	p<0.01	p<0.05	p>0.05	p>0.05	0.12	61.63	4.21
**Occipital Lobe**												
BA17	1.7e-01	p<0.001	-1.6e-03	p<0.001	-3.92E-04	p>0.05	p>0.05	p>0.05	p>0.05	0.05	52.57	2.69
BA18	1.1e-01	p<0.01	-1e-03	p<0.01	-3.61E-04	p>0.05	p>0.05	p>0.05	p>0.05	0.02	52.38	2.76
BA19	3.7e-02	p>0.05	-3.4e-04	p>0.05	9.77e-05	p>0.05	p>0.05	p>0.05	p>0.05	-	-	-
**Mixed**												

BA26	2.2e-01	p>0.05	-2e-03	p>0.05	3.29e-04	p>0.05	p>0.05	p>0.05	p>0.05	-	-	-
BA27	2.6e-01	p<0.01	-2.7e-03	p<0.01	-5.97E-04	p>0.05	p<0.01	p>0.05	p>0.05	0.06	48.48	2.84
BA28	1.3e-01	p>0.05	-1.6e-03	p<0.05	-5.97E-04	p>0.05	p>0.05	p>0.05	p>0.05	0.05	40.82	5.28
BA35	2.9e-01	p<0.01	-3e-03	p<0.01	-5.93E-04	p>0.05	p>0.05	p>0.05	p>0.05	0.05	48.48	2.47
BA36	2.2e-01	p<0.001	-2.2e-03	p<0.001	-1.28E-03	p<0.001	p>0.05	p>0.05	p>0.05	0.08	51.31	2.05
BA43	4.9e-02	p>0.05	-7.9e-04	p>0.05	-4.50E-04	p>0.05	p>0.05	p<0.05	p>0.05	-	-	-
**Susceptibility--WM bundle**												
ARCL	-2.2e-01	p<0.0001	2.4e-03	p<0.0001	-5.40E-05	p>0.05	p>0.05	p>0.05	p>0.05	0.09	47.36	1.61
ARCR	-2.7e-01	p<0.0001	2.9e-03	p<0.0001	3.69e-04	p>0.05	p>0.05	p>0.05	p<0.05	0.15	47.14	1.49
ATRL	-1.9e-01	p<0.001	2.1e-03	p<0.0001	4.60e-04	p>0.05	p>0.05	p>0.05	p>0.05	0.08	43.69	3.08
ATRR	-2.1e-01	p<0.0001	2.3e-03	p<0.0001	8.64e-04	p<0.01	p>0.05	p>0.05	p>0.05	0.13	45.12	2.02
CGCL	3.3e-02	p>0.05	-6e-04	p>0.05	-1.95E-04	p>0.05	p>0.05	p>0.05	p>0.05	-	-	-
CGCR	1.6e-02	p>0.05	-8.6e-05	p>0.05	-2.74E-04	p>0.05	p>0.05	p>0.05	p>0.05	-	-	-
CSTL	-1.9e-01	p<0.0001	1.8e-03	p<0.0001	2.85e-04	p>0.05	p>0.05	p>0.05	p>0.05	0.07	53.70	2.45
CSTR	-2e-01	p<0.0001	2.1e-03	p<0.0001	5.92e-04	p<0.05	p>0.05	p>0.05	p>0.05	0.06	49.59	2.31
FA	-2e-01	p<0.01	2.6e-03	p<0.001	9.15e-04	p<0.05	p>0.05	p>0.05	p>0.05	0.15	37.78	3.55
FP	-7.2e-02	p>0.05	1.3e-03	p>0.05	-2.33E-04	p>0.05	p>0.05	p>0.05	p>0.05	-	-	-
IFOL	-7.2e-02	p>0.05	7.1e-04	p>0.05	-1.98E-05	p>0.05	p>0.05	p>0.05	p>0.05	-	-	-
IFOR	-1.9e-01	p<0.01	1.9e-03	p<0.01	1.00e-03	p<0.01	p>0.05	p>0.05	p>0.05	0.05	50.30	2.56
ILFL	-3.6e-02	p>0.05	7.3e-04	p>0.05	4.24e-05	p>0.05	p<0.05	p>0.05	p>0.05	-	-	-
ILFR	-1.7e-01	p<0.05	2.1e-03	p<0.01	6.68e-04	p>0.05	p>0.05	p>0.05	p<0.05	0.09	40.78	3.55
pARCL	-3.5e-01	p<0.0001	3.7e-03	p<0.0001	-3.25E-04	p>0.05	p>0.05	p>0.05	p>0.05	0.13	46.97	1.49
pARCR	-2.8e-01	p<0.001	3e-03	p<0.0001	-2.64E-04	p>0.05	p>0.05	p>0.05	p>0.05	0.09	45.64	2.28
SLFL	-2.6e-01	p<0.0001	2.6e-03	p<0.0001	1.34e-04	p>0.05	p>0.05	p>0.05	p>0.05	0.10	49.78	1.38
SLFR	-3.1e-01	p<0.0001	3.2e-03	p<0.0001	2.20e-04	p>0.05	p>0.05	p>0.05	p<0.05	0.15	49.41	1.16
VOFR	-3e-01	p<0.0001	3e-03	p<0.0001	-1.95E-04	p>0.05	p>0.05	p>0.05	p<0.05	0.09	48.69	2.29
VOFL	-1.1e-01	p>0.05	1.2e-03	p>0.05	-6.90E-04	p>0.05	p>0.05	p>0.05	p<0.05	-	-	-
UNCR	-1.6e-01	p<0.01	1.3e-03	p<0.05	1.77e-04	p>0.05	p>0.05	p>0.05	p>0.05	-	-	-
UNCL	-8.1e-02	p>0.05	3.8e-04	p>0.05	-7.74E-04	p<0.05	p>0.05	p>0.05	p>0.05	-	-	-

Abbreviation: SD: standard deviation; cGM: cortical grey matter; sWM: superficial white matter; WM: white matter; FA: Callosum Forceps Minor; FP: Callosum Forceps Major; ARC: Arcuate; pARC: Posterior Arcuate Fasciculus; ATR: Thalamic Radiation; CGC: Cingulum Cingulate; CST: Corticospinal; IFO: Inferior Fronto-Occipital Fasciculus; ILF: Inferior Longitudinal Fasciculus; SLF: Superior Longitudinal Fasciculus; VOF: Vertical Occipital Fasciculus; UNC: Uncinate; L: left; R: right; BA: Brodmann Area.

Supplementary Table 3: Model evaluation analysis

Region	Bspline EXPV	Polynomial EXPV	EXPV Ratio (Bspline/Polynomial)
**R1-cGM**			
**Frontal Lobe**			
BA4	0.45	0.53	0.86
BA6	0.57	0.56	1.03
BA8	0.56	0.64	0.88
BA9	0.55	0.54	1.01
BA10	0.42	0.46	0.91
BA11	0.48	0.52	0.93
BA24	0.60	0.60	1.00
BA25	0.43	0.41	1.04
BA32	0.49	0.48	1.02
BA33	0.35	0.38	0.94
BA44	0.55	0.62	0.90
BA45	0.48	0.50	0.96
BA46	0.61	0.51	1.21
BA47	0.51	0.61	0.83
**Parietal Lobe**			
BA1	0.42	0.46	0.93
BA2	0.44	0.56	0.79
BA3	0.51	0.35	1.48
BA5	0.48	0.46	1.04
BA7	0.46	0.45	1.01
BA23	0.40	0.51	0.79
BA29	0.41	0.45	0.92
BA30	0.39	0.36	1.09
BA31	0.48	0.46	1.05
BA39	0.42	0.46	0.92
BA40	0.54	0.47	1.15
**Temporal Lobe**			
BA20	0.47	0.57	0.82
BA21	0.50	0.44	1.13
BA22	0.51	0.60	0.84
BA37	0.42	0.38	1.11
BA38	0.41	0.51	0.79
BA41	0.40	0.44	0.91
BA42	0.41	0.42	0.98
**Occipital Lobe**			
BA17	0.27	0.19	1.40
BA18	0.32	0.39	0.81
BA19	0.41	0.40	1.04
**Mixed**	-	-	-
BA26	0.15	0.23	0.67
BA27	0.35	0.38	0.92
BA28	0.39	0.42	0.92
BA35	0.35	0.37	0.94
BA36	0.50	0.39	1.26
BA43	0.42	0.48	0.89
**R1-sWM**			
**Frontal Lobe**			
BA4	0.52	0.49	1.06
BA6	0.47	0.49	0.98
BA8	0.33	0.33	1.01
BA9	0.43	0.43	1.00
BA10	0.45	0.45	1.00
BA11	0.36	0.40	0.91
BA24	0.44	0.38	1.16
BA25	0.21	0.20	1.08
BA32	0.37	0.41	0.92
BA33	0.46	0.47	0.97
BA44	0.50	0.45	1.11
BA45	0.44	0.48	0.92
BA46	0.46	0.50	0.93
BA47	0.42	0.35	1.20
**Parietal Lobe**			

BA1	0.29	0.30	0.98
BA2	0.37	0.35	1.06
BA3	0.23	0.39	0.58
BA5	0.45	0.44	1.03
BA7	0.38	0.40	0.95
BA23	0.46	0.39	1.20
BA29	0.22	0.20	1.08
BA30	0.36	0.37	0.97
BA31	0.44	0.42	1.05
BA39	0.35	0.48	0.72
BA40	0.46	0.51	0.89
**Temporal Lobe**			
BA20	0.32	0.38	0.83
BA21	0.47	0.36	1.30
BA22	0.32	0.27	1.17
BA37	0.42	0.40	1.04
BA38	0.38	0.46	0.83
BA41	0.49	0.36	1.37
BA42	0.50	0.44	1.13
**Occipital Lobe**			
BA17	0.23	0.29	0.81
BA18	0.51	0.35	1.46
BA19	0.42	0.40	1.04
**Mixed**	-	-	-
BA26	0.24	0.24	1.01
BA27	0.19	0.17	1.17
BA28	0.22	0.21	1.09
BA35	0.30	0.33	0.91
BA36	0.19	0.18	1.04
BA43	0.37	0.38	0.97
**R1-WM bundles**			
ARCL	0.39	0.46	0.85
ARCR	0.36	0.44	0.80
ATRL	0.45	0.45	1.00
ATRR	0.39	0.52	0.75
CGCL	0.49	0.37	1.35
CGCR	0.41	0.37	1.13
CSTL	0.48	0.52	0.93
CSTR	0.39	0.50	0.78
FA	0.54	0.50	1.08
FP	0.44	0.49	0.90
IFOL	0.54	0.50	1.07
IFOR	0.52	0.49	1.06
ILFL	0.52	0.47	1.09
ILFR	0.46	0.47	0.97
pARCL	0.33	0.46	0.72
pARCR	0.43	0.31	1.38
SLFL	0.46	0.39	1.19
SLFR	0.46	0.52	0.89
VOFR	0.45	0.46	0.98
VOFL	0.41	0.42	0.97
UNCR	0.28	0.22	1.30
UNCL	0.19	0.19	0.97
**R2*-cGM**			
**Frontal Lobe**			
BA4	0.48	0.44	1.10
BA6	0.52	0.54	0.97
BA8	0.42	0.46	0.92
BA9	0.36	0.50	0.72
BA10	0.32	0.30	1.08
BA11	0.39	0.52	0.76
BA24	0.39	0.38	1.05
BA25	0.33	0.30	1.12
BA32	0.39	0.38	1.03
BA33	0.22	0.31	0.72
BA44	0.54	0.41	1.34
BA45	0.36	0.33	1.12

BA46	0.44	0.40	1.09
BA47	0.40	0.36	1.09
**Parietal Lobe**			
BA1	0.35	0.28	1.26
BA2	0.44	0.36	1.20
BA3	0.27	0.18	1.50
BA5	0.27	0.27	0.98
BA7	0.35	0.36	0.97
BA23	0.29	0.19	1.54
BA29	-	-	-
BA30	0.25	0.24	1.00
BA31	0.26	0.24	1.06
BA39	0.32	0.31	1.05
BA40	0.37	0.39	0.96
**Temporal Lobe**			
BA20	0.34	0.44	0.78
BA21	0.33	0.39	0.84
BA22	0.43	0.58	0.75
BA37	0.35	0.39	0.91
BA38	0.33	0.41	0.81
BA41	0.31	0.28	1.12
BA42	0.44	0.41	1.07
**Occipital Lobe**			
BA17	0.22	0.27	0.81
BA18	0.36	0.28	1.28
BA19	0.28	0.33	0.86
**Mixed**			
BA26	-	-	-
BA27	0.31	0.27	1.16
BA28	0.26	0.34	0.76
BA35	0.21	0.22	0.97
BA36	0.34	0.34	1.00
BA43	0.35	0.35	1.00
**R2*-sWM**			
**Frontal Lobe**			
BA4	0.30	0.39	0.77
BA6	0.28	0.34	0.82
BA8	0.44	0.31	1.41
BA9	0.37	0.34	1.08
BA10	0.35	0.31	1.13
BA11	0.25	0.21	1.17
BA24	0.32	0.31	1.04
BA25	0.23	0.24	0.96
BA32	0.36	0.28	1.29
BA33	0.28	0.45	0.64
BA44	0.29	0.42	0.68
BA45	0.34	0.25	1.37
BA46	0.33	0.31	1.06
BA47	0.24	0.29	0.84
**Parietal Lobe**			
BA1	0.32	0.47	0.69
BA2	0.30	0.27	1.11
BA3	0.31	0.35	0.88
BA5	0.37	0.26	1.41
BA7	0.32	0.29	1.10
BA23	0.26	0.21	1.24
BA29	0.29	0.20	1.45
BA30	0.16	0.20	0.78
BA31	0.23	0.29	0.80
BA39	0.30	0.37	0.82
BA40	0.30	0.30	0.98
**Temporal Lobe**			
BA20	0.27	0.27	1.00
BA21	0.29	0.42	0.71
BA22	0.43	0.40	1.07
BA37	0.23	0.29	0.78
BA38	0.28	0.30	0.93

BA41	0.33	0.29	1.14
BA42	0.33	0.36	0.90
**Occipital Lobe**			
BA17	0.15	0.24	0.65
BA18	0.36	0.30	1.18
BA19	0.28	0.28	1.02
**Mixed**			
BA26	0.25	0.35	0.72
BA27	0.30	0.28	1.07
BA28	0.42	0.30	1.39
BA35	0.22	0.19	1.19
BA36	0.37	0.30	1.26
BA43	0.27	0.34	0.79
**R2*-WM bundles**		-	-
ARCL	0.22	0.31	0.71
ARCR	0.21	0.31	0.69
ATRL	0.31	0.40	0.78
ATRR	0.31	0.35	0.87
CGCL	0.31	0.31	0.99
CGCR	0.23	0.19	1.25
CSTL	0.17	0.26	0.68
CSTR	0.27	0.32	0.84
FA	0.25	0.26	0.99
FP	0.17	0.14	1.21
IFOL	0.26	0.29	0.88
IFOR	0.29	0.27	1.05
ILFL	0.32	0.44	0.74
ILFR	0.20	0.26	0.78
pARCL	0.24	0.18	1.32
pARCR	0.27	0.22	1.27
SLFL	0.26	0.24	1.08
SLFR	0.16	0.34	0.47
VOFR	0.17	0.34	0.48
VOFL	0.24	0.34	0.71
UNCR	0.23	0.32	0.73
UNCL	0.25	0.28	0.91
**Susceptibility-cGM**			
**Frontal Lobe**			
BA4	0.28	0.42	0.66
BA6	-0.08	-0.07	1.07
BA8	0.14	0.13	1.09
BA9	-0.10	-0.12	0.81
BA10	-	-	-
BA11	-	-	-
BA24	0.13	0.23	0.58
BA25	-	-	-
BA32	0.25	0.15	1.70
BA33	-	-	-
BA44	0.22	0.12	1.80
BA45	0.16	0.24	0.64
BA46	0.15	0.25	0.61
BA47	-	-	-
**Parietal Lobe**			
BA1	0.27	0.26	1.01
BA2	0.35	0.27	1.30
BA3	-	-	-
BA5	0.23	0.16	1.42
BA7	0.16	0.25	0.64
BA23	0.37	0.26	1.43
BA29	-	-	-
BA30	0.11	0.13	0.88
BA31	0.29	0.25	1.19
BA39	0.16	0.20	0.82
BA40	-0.08	-0.09	0.93
**Temporal Lobe**			
BA20	-	-	-
BA21	-	-	-

BA22	0.18	0.15	1.22
BA37	0.22	0.14	1.58
BA38	-	-	-
BA41	0.29	0.31	0.94
BA42	0.14	0.22	0.65
**Occipital Lobe**			
BA17	0.26	0.29	0.89
BA18	0.13	0.10	1.34
BA19	0.31	0.21	1.46
**Mixed**	-	-	-
BA26	-	-	-
BA27	-	-	-
BA28	-	-	-
BA35	-	-	-
BA36	-	-	-
BA43	0.30	0.34	0.91
**Susceptibility-sWM**			
**Frontal Lobe**			
BA4	-	-	-
BA6	0.24	0.15	1.64
BA8	0.24	0.31	0.77
BA9	0.19	0.23	0.86
BA10	0.35	0.21	1.64
BA11	-	-	-
BA24	0.10	0.10	1.06
BA25	0.25	0.15	1.61
BA32	0.18	0.17	1.01
BA33	0.17	0.22	0.78
BA44	0.16	0.23	0.67
BA45	-	-	-
BA46	0.27	0.26	1.04
BA47	-	-	-
**Parietal Lobe**			
BA1	-	-	-
BA2	0.18	0.15	1.15
BA3	-	-	-
BA5	0.15	0.22	0.68
BA7	-	-	-
BA23	0.30	0.32	0.94
BA29	-	-	-
BA30	-	-	-
BA31	0.08	0.22	0.38
BA39	-	-	-
BA40	-	-	-
**Temporal Lobe**			
BA20	-	-	-
BA21	-	-	-
BA22	-	-	-
BA37	-	-	-
BA38	-	-	-
BA41	-	-	-
BA42	0.33	0.32	1.04
**Occipital Lobe**			
BA17	0.14	0.22	0.64
BA18	0.08	0.11	0.73
BA19	-	-	-
**Mixed**	-	-	-
BA26	-	-	-
BA27	-0.13	-0.11	1.20
BA28	0.10	0.09	1.05
BA35	0.08	0.10	0.80
BA36	0.18	0.19	0.98
BA43	-	-	-
**Susceptibility-WM bundles**			
ARCL	0.16	0.27	0.58
ARCR	0.35	0.28	1.25
ATRL	0.20	0.21	0.97

ATRR	0.25	0.26	0.97
CGCL	-	-	-
CGCR	-	-	-
CSTL	0.22	0.26	0.87
CSTR	0.14	0.26	0.52
FA	0.22	0.28	0.81
FP	-	-	-
IFOL	-	-	-
IFOR	0.29	0.36	0.82
ILFL	-	-	-
ILFR	0.22	0.25	0.89
pARCL	0.06	0.06	0.92
pARCR	0.27	0.37	0.72
SLFL	0.20	0.20	0.97
SLFR	0.19	0.20	0.94
VOFR	0.12	0.14	0.85
VOFL	-	-	-
UNCR	0.23	0.26	0.87
UNCL	-	-	-

Abbreviation: SD: standard deviation; cGM: cortical grey matter; sWM: superficial white matter; WM: white matter; FA: Callosum Forceps Minor; FP: Callosum Forceps Major; ARC: Arcuate; pARC: Posterior Arcuate Fasciculus; ATR: Thalamic Radiation; CGC: Cingulum Cingulate; CST: Corticospinal; IFO: Inferior Fronto-Occipital Fasciculus; ILF: Inferior Longitudinal Fasciculus; SLF: Superior Longitudinal Fasciculus; VOF: Vertical Occipital Fasciculus; UNC: Uncinate; L: left; R: right; BA: Brodmann Area.

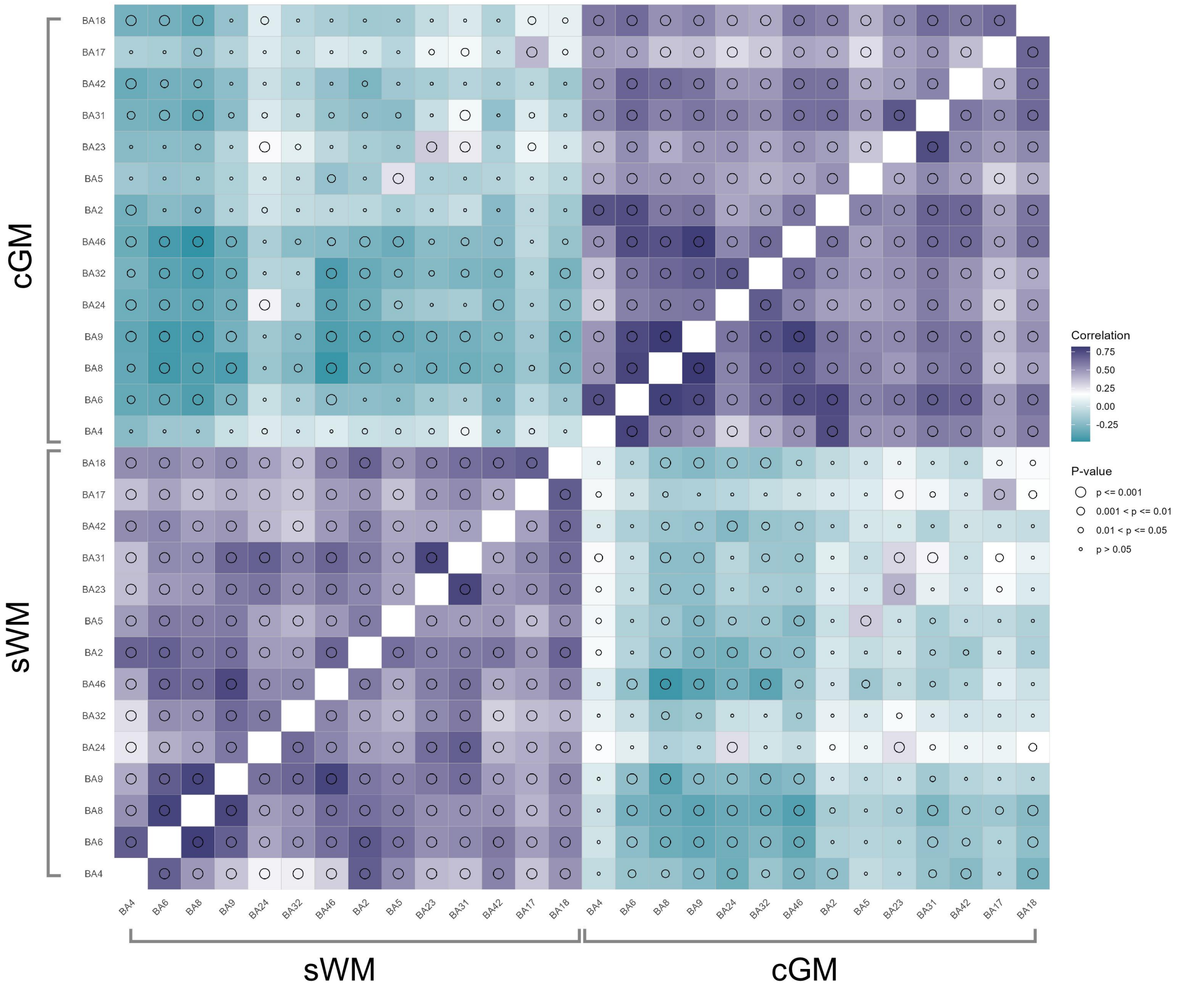

Supplementary Figure 1: Quantitative Correlation Analysis in QSM

This figure shows the Pearson correlation matrix for susceptibility values, illustrating intra- and inter-regional patterns within and between cGM and sWM. In each matrix, the upper triangles depict Z-scores derived from the residuals of significant quadratic models adjusted for ageing effects, illustrating correlations that are independent of age. The lower triangles display the raw average values for each region, highlighting the overall connectivity. The circle size increases as the P-value decreases, and darker colours indicate stronger correlations. Only regions with significant quadratic regression fits were included in the matrix calculations. Each submatrix is subdivided into five lobes based on the anatomical locations of the selected Brodmann areas. Region labels indicate the order and anatomical position of each region within the matrix. Pearson correlation coefficients (r) with corresponding P-values are shown. The experimental unit was the individual participant (N = 293). cGM: cortical grey matter; sWM: superficial white matter; BA: Brodmann area; QSM: quantitative susceptibility mapping.

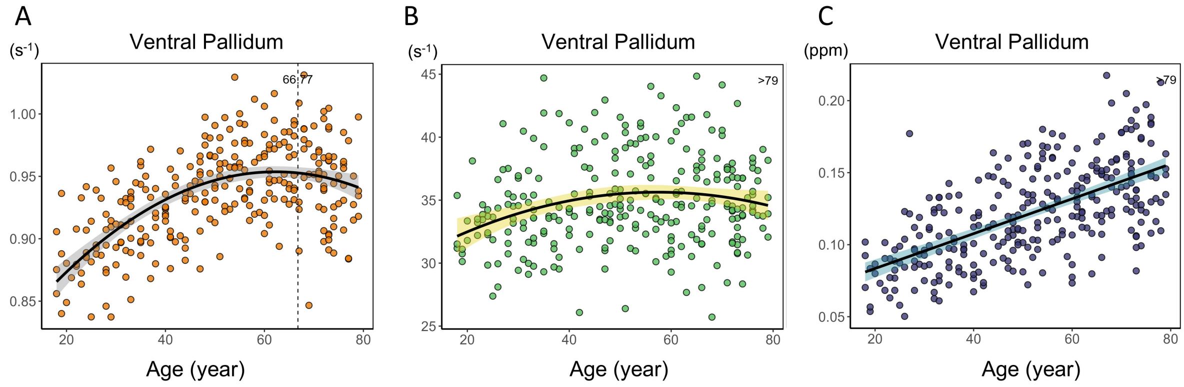

Supplementary Figure 2: Normative Age Modelling in the Ventral Pallidum

This figure presents the normative age modelling of quantitative MRI metrics within the ventral pallidum, a deep grey matter region characterised by high iron content. A–C: Fitting models for (A) R_1, (B) R_2*, and (C) Quantitative Susceptibility Mapping (QSM), respectively. Each scatter plot represents individual participants (N = 293). Quadratic polynomial regression models (solid lines) were fitted for R_1 and R_2* to capture nonlinear age-related changes, while a linear model was fitted for QSM based on likelihood ratio testing ($P < 0.05$). Shaded areas indicate 95% confidence intervals derived from bootstrap resampling (N = 10000). The R_1 quadratic model showed a turning point at 66.77 years, whereas that of R_2* extended beyond the maximum cohort age (79 years), with an estimated peak at 98.92 years. QSM values exhibited a steady linear increase with age. QSM: Quantitative Susceptibility Mapping.

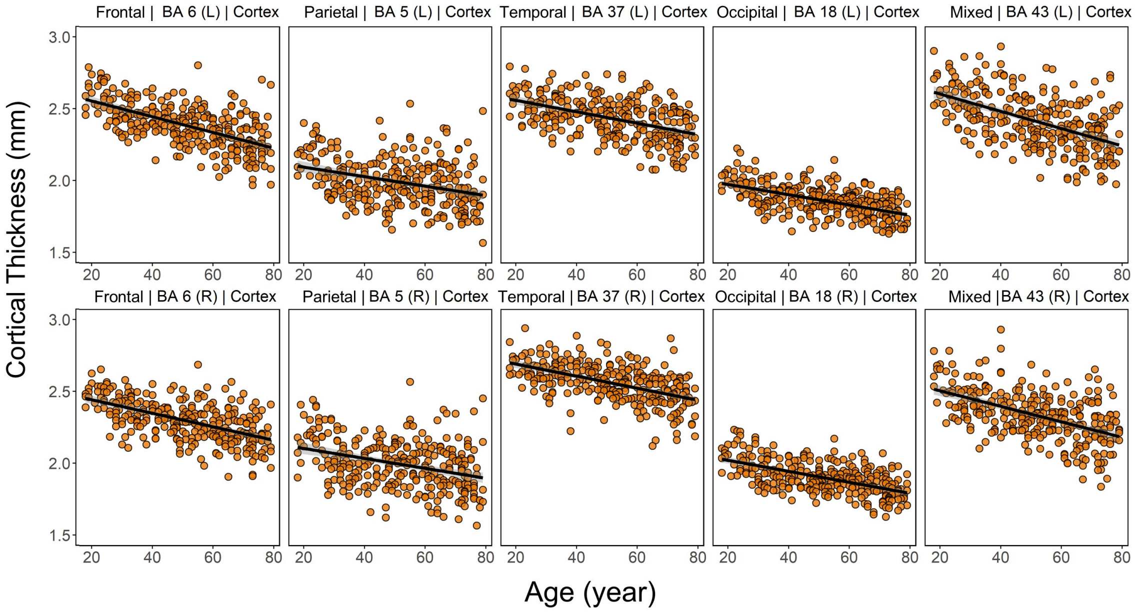

Supplementary Figure 3: Normative Age Modelling of Cortical Thickness

This figure presents normative age modelling of cortical thickness across representative cortical grey matter regions in different brain lobes. Each scatter plot represents individual participants (N = 293). Cortical thickness values were modelled as a function of age, additionally with intracranial volume included as a covariate to account for inter-individual differences in head size. Linear and quadratic models were compared using likelihood ratio tests, and linear fits (solid lines) were generally preferred ($P < 0.05$). Shaded areas indicate 95% confidence intervals derived from bootstrap resampling (N = 10,000). BA: Brodmann Area; L: Left Hemisphere; R: Right Hemisphere.
